# An Engineered Biarylitide Cross-Linking P450 from RiPP Biosynthesis Generates Alternative Cyclic Peptides

**DOI:** 10.1021/acs.orglett.3c04366

**Published:** 2024-02-28

**Authors:** Maxine Treisman, Laura Coe, Yongwei Zhao, Vishnu Mini Sasi, Jemma Gullick, Mathias H. Hansen, Aviva Ly, Victor Leichthammer, Caroline Hess, Daniel L. Machell, Ralf B. Schittenhelm, Joel Hooper, Colin J. Jackson, Julien Tailhades, James J. De Voss, Max J. Cryle

**Affiliations:** † Department of Biochemistry and Molecular Biology, The Monash Biomedicine Discovery Institute, Monash University, EMBL Australia, Clayton, VIC 3800, Australia; ‡ ARC Centre of Excellence for Innovations in Peptide and Protein Science, Clayton, VIC 3800, Australia; § School of Chemistry and Molecular Biosciences, The University of Queensland, Brisbane, QLD 4072, Australia; ∥ Research School of Chemistry, The Australian National University, Acton, ACT 2601, Australia; ⊥ Department of Chemistry, Monash University, Clayton, VIC 3800, Australia; # Monash Proteomics and Metabolomics Platform, Monash University, Clayton, VIC 3800, Australia; ∇ ARC Centre of Excellence in Synthetic Biology, Australian National University, Acton, ACT 2601, Australia; % Research School of Biology, Australian National University, Acton, ACT 2601, Australia

## Abstract

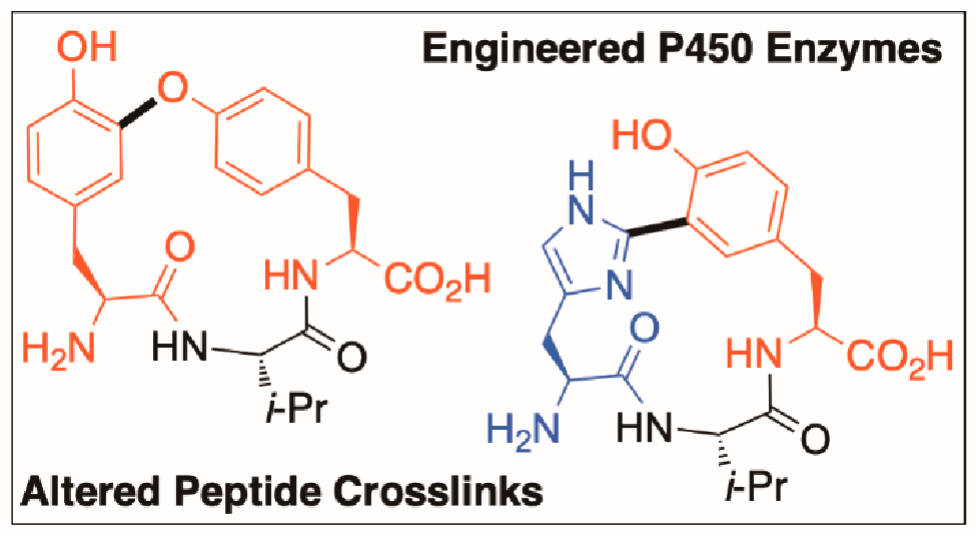

Cytochrome-P450-mediated cross-linking of ribosomally encoded peptides (RiPPs) is rapidly expanding and displays great potential for biocatalysis. Here, we demonstrate that active site engineering of the biarylitide cross-linking enzyme P450_Blt_ enables the formation of His-X-Tyr and Tyr-X-Tyr cross-linked peptides, thus showing how such P450s can be further exploited to produce alternate cyclic tripeptides with controlled cross-linking states.

Peptide natural products are capable of highly diverse activity and find application across a range of discovery, industrial, agricultural and medical applications.^1^ One common and important feature of peptides is cyclization, which enables improved activity and metabolic stability.^2^ This process can occur through a range of biosynthetic processes including disulfide bond formation, head/side chain to tail cyclization, or the side chain cross-linking of residues within these peptides. While non-ribosomal peptides remain arguably the most important class of bioactive peptides, peptides produced by ribosomal pathways (RiPPs) are growing in importance, both from the bioactivity they display and the potential that such pathways exhibit for the engineered biosynthesis of novel peptides at scale.^2^ This makes the identification and characterization of key enzymes involved in RiPP pathways of high importance due to their potential to act as biocatalysts, both naturally and as engineered catalysts.^2^

One important family of powerful biosynthetic enzymes is the cytochromes P450 (P450s), which are widespread within secondary metabolism due to the diverse range of biosynthetic transformations that they can catalyze.^3^ While the archetypal P450-catalyzed reaction is the hydroxylation of unactivated C–H bonds, P450s also play important roles in the biosynthesis of complex peptide macrocycles, often through the specific oxidative cross-linking of aromatic residues found within such peptides.^3^ Key examples of these processes include the biosynthesis of the non-ribosomal peptides arylomycin and the glycopeptide antibiotics (including vancomycin and teicoplanin),^4−6^ along with more recent examples found in the biosynthesis of various families of RiPPs (Figure 1).^7−10^

**Figure 1:**
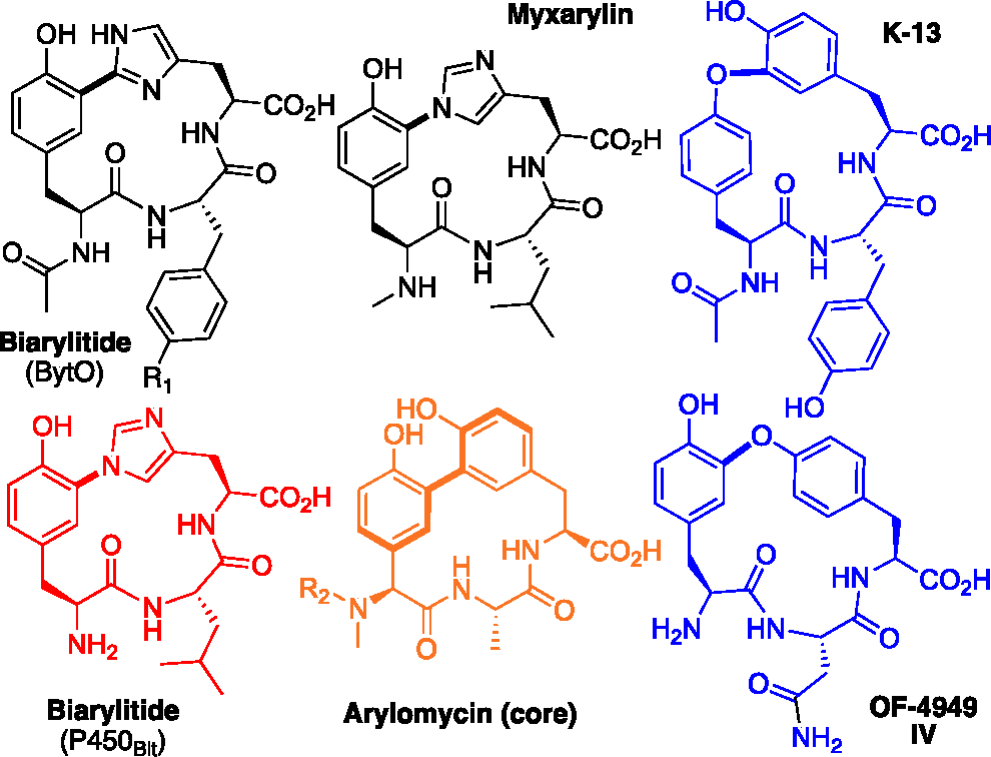
Cytochrome-P450-mediated cross-linking forming cyclic tripeptides from ribosomal pathways (P450_Blt_ in red) and related natural products of NRPS (orange) and unknown biosynthetic origins (blue). R = H/OH and R_2_ = fatty acyl-(D)NMe-Ser-(D)-Ala-Gly-.

These systems are important to study as model systems to further understand such powerful biotransformations given the relative simplicity of these systems in comparison to carrier protein bound substrates typically found in non-ribosomal peptide biosynthesis, while also being of interest themselves as potential biocatalysts for peptide synthesis. Among RiPP pathways, the P450 enzymes that generate the biarylitides (and related myxarylins)^11^ have attracted considerable interest as potential biocatalysts for the generation of Tyr-X-His and Tyr-X-Trp cyclic tripeptides as they require limited leader peptide sequences and tolerate altered peptide substrates.^10,12^ Furthermore, extensive characterization of key P450s from biarylitide pathways provides significant insights into how such enzymes bind and oxidize their peptide substrates, enabling the engineering of such enzymes.^10,13^ In this study, we reveal how to engineer biarylitide P450s as biocatalysts to generate novel His-X-Tyr and Tyr-X-Tyr cross-linked tripeptides, demonstrating their potential as high value biocatalysts for peptide biosynthesis.

We commenced our study by engineering the P450 enzyme P450_Blt_ from *Micromonospora* sp. MW-13, which we have previously demonstrated accepts minimal pentapeptide substrates with the sequence MRYXH and where X can encode considerable side chain diversity (native substrate **1**: MRYLH).^10^ Our choice of substrates for these engineered P450s (Scheme 1, Supporting Information (SI) Figures S1–S18) included a reversed His-X-Tyr substrate (**2**: MRHLY) and Tyr-X-Tyr substrate (**3**: MRYLY), with neither being an effective substrate for the wildtype P450_Blt_ enzyme (**2**: < 2%, **3**: 29%). Through analysis of the structure of the MRYLH bound P450 structure and the mechanistic implications of active site residues and inspection of P450s known to encode the cross-linking of altered pentapeptide substrates, we identified the I-helix residues A231, H234, and E238 as of key importance in controlling the specificity of P450_Blt_.^13^ We had previously characterized the activity of related single mutants at these positions toward **1**, which showed different degrees of reduced activity in all cases (E238A was prepared in lieu of E238N which was insoluble).^13^ Given this, we postulated that mutations at two of these positions would be required to improve the activity of P450_Blt_ toward novel substrates. Indeed, the conversions of **2** and **3** with these single mutants showed limited activity, although the activity of the A231V mutant toward **2** had been encouraging.^13^

**Scheme 1:**
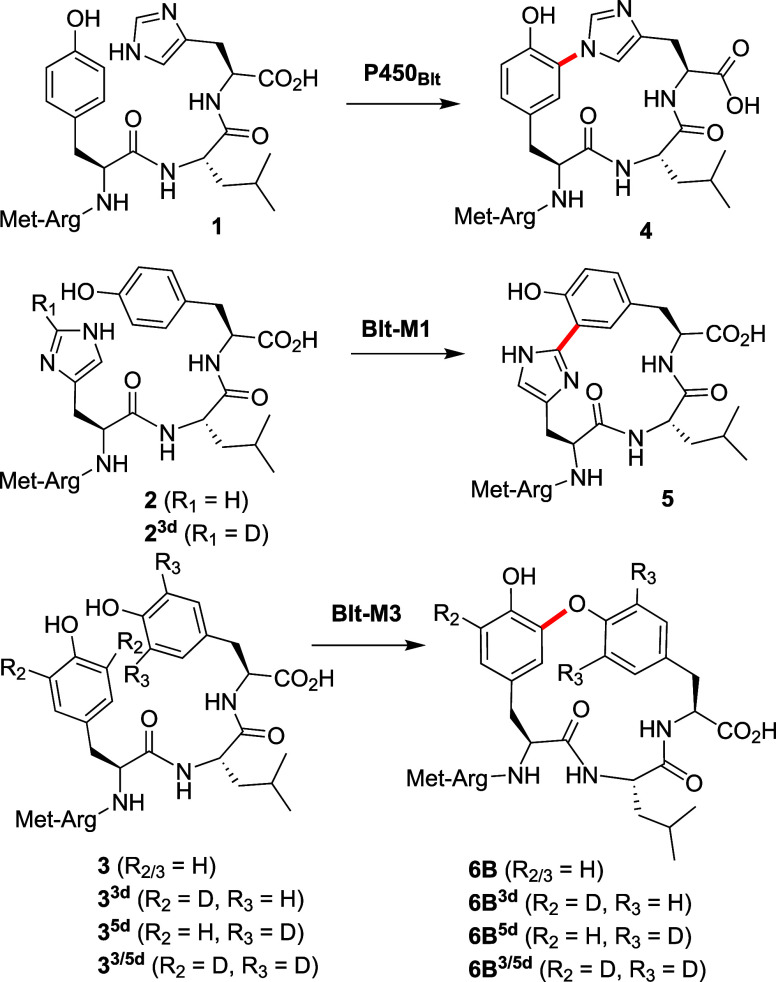
Linear Peptide Substrates (**1**–**3**) Explored in This Study Together with the Products of P450_Blt_ (WT and Mutant)-Mediated Cyclization (**4**–**6**) Deuterium labeled peptides are named with the superscript indicating the residue of the peptide that is deuterated.

Thus, we generated 3 double mutants in which these positions were altered to the sequence of a P450 proposed to prefer a reversed His-X-Tyr substrate (MRHEY; *Blt-M1*: A231V-H234L, *Blt-M2*: A231V-E238N, *Blt-M3*: H234L-E238N)^12^ and explored their activity toward **1**–**3** (Table 1). We first quantified the effect of these mutations on peptide affinity for these mutants (Table 1, SI Figure S19) and identified that *Blt-M1* and *Blt-M3* showed affinities for **1**–**3** comparable to the wildtype enzyme for **1**;^10^ importantly, these mutants showed high affinity for **2** and **3**, which was not observed from the WT enzyme with these altered substrates. The affinities of **1**–**3** for *Blt-M2* were all reduced by at least an order of magnitude compared to the binding of **1** by the WT enzyme, which matched the low levels of conversion for all substrates by this mutant. Turning next to *Blt-M1*, we were excited to see high levels of cyclization for both **2** and **3** affording **5** and **6**, respectively (≥70%, Table 1), with concomitant reduction in cyclization of **1**. With similar affinity binding seen for *Blt-M3*, we anticipated comparable levels of cyclization for **1**–**3**, although here high activity (∼85%, Table 1) was only now observed for **3** (affording **6**).

**Table 1:** Binding Affinity and Enzymatic Cyclization of **1**–**3** by P450_Blt_ Double Mutants M1–M3

Enzyme	Substrate (Product)	Binding^b^	*K*_D_ (μM)	Conversion (%)
P450_Blt_^10^	**1**	Type I	2.1	85
	**2**	Type I	Weak	>2% ± 1
	**3**	Type I	Weak	29 ± 3
Blt-M1	**1 (4)**	Type I	1.1 ± 0.6	20 ± 18
	**2 (5)**	Type I	1.4 ± 1	76 ± 3
	**3 (6)**	Type I	1.6 ± 0.9	70 ± 6
Blt-M2^a^	**1**	Type I	17	14
	**2**	Type I	61	17
	**3**	Hybrid	32	27
Blt-M3	**1**	Type II	3.3 ± 2.6	9 ± 3
	**2**	Type I	4.3 ± 4.2	8 ± 7
	**3**	Type II	1.7 ± 0.7	85 ± 3

a Single measurements due to the low expression yield of M2.

b See SI Figure S19.

Given that there are multiple cross-links possible for **6** ((Tyr_3_C-Tyr_5_C (**6A**), Tyr_3_C-Tyr_5_O (**6B**), and Tyr_3_O-Tyr_5_C (**6C**)) that are comparable to the cross-linking patterns found naturally in arylomycin,^14^ OF-4949,^15^ and K-13, respectively,^16^ we then explored the activity of P450_Blt_ and mutants toward **3**. Of these enzymes *Blt-M3* was the most selective for formation of a single product (>90%), and we scaled the *Blt-M3* cross-linking reaction to identify the cross-link formed (Figure 2A). Using a combination of 1D and 2D NMR we confirmed the structure of this cyclic tripeptide as containing a C_3_–O_5_ cross-link **6B**, which is the same cross-link type as is found in the natural product OF-4949 (Figure 2C,D). The ^1^H NMR spectrum exhibited key features consistent with the pentapeptide **6**: 5 α protons, 4 amide NH protons, and 7 aromatic Hs, suggestive of a TyrC–TyrO cross-link. ^13^C-HSQC and ^15^N-HSQC spectra allowed the corresponding carbon and nitrogen resonances to be identified. Full structural assignment was completed using 2D COSY, TOCSY, HMBC, and NOESY experiments. A single sharp proton signal was identified at δ_H_ 9.09 with no corresponding cross-peak in the ^13^C-HSQC or ^15^N-HSQC spectra. This was identified as a free Tyr phenolic group, again consistent with a TyrC–TyrO cross-link. Importantly, four carbonyl carbons were located by analysis of the ^13^C-HMBC spectrum at δ_c_ 170.4, 168.3, 171.0, and 173.0. The seven aromatic proton signals displayed the coupling expected for one *para*-disubstituted and one trisubstituted Tyr residue. The phenolic proton at δ_H_ 9.09 demonstrated HMBC correlations to C6″ (δ_c_ 147.4), C7″ (δ_c_ 145.0), and C8″ (δ_c_ 115.8). Analyses of the HMBC and COSY correlations (Figure 2D, SI Figures S20–S25) were consistent with the cross-link positioned at C6″, *ortho* to the phenolic position C7″. The C6″ chemical shift is that expected for a carbon in a C–O–C bond and compares favorably to the range reported for such carbons in the OF-4949 compound family, δ_c_ 145–148 (ammonium-*d*_4_ deuteroxide).^15^ In K13, the analogous position is reported at δ_c_ 148 (methanol-*d*_4_).^16^ This chemical shift can be contrasted with that seen in arylomycin A_2_, where the C–C tyrosine linkage results in a resonance significantly further upfield at δ_c_ 125.4 (DMSO-*d*_6_).^14^ The signals associated with the *para*-disubstituted Tyr residue consisted of four chemically inequivalent protons rather than the A_2_X_2_ type system typically observed for a linear tyrosine residue.^16^ This observation is consistent with reported data for OF-4949.^15^ HMBC correlations were observed from these four aromatic positions to the C7⁗ ether carbon (δ_c_ 153.4). Notably the amide carbonyl C1″ (δ_c_ 168.3) displayed HMBC correlations to both the α- and β-position protons of the Tyr residue that possesses the free phenolic position. Carbonyl C1″ is also correlated to the Leu α-proton H2‴ (Figure 2D). These observations confirm a Tyr_3_C–Tyr_5_O (**6B**) cross-link and are inconsistent with a Tyr_3_O–Tyr_5_C (**6C**) cross-link.

**Figure 2:**
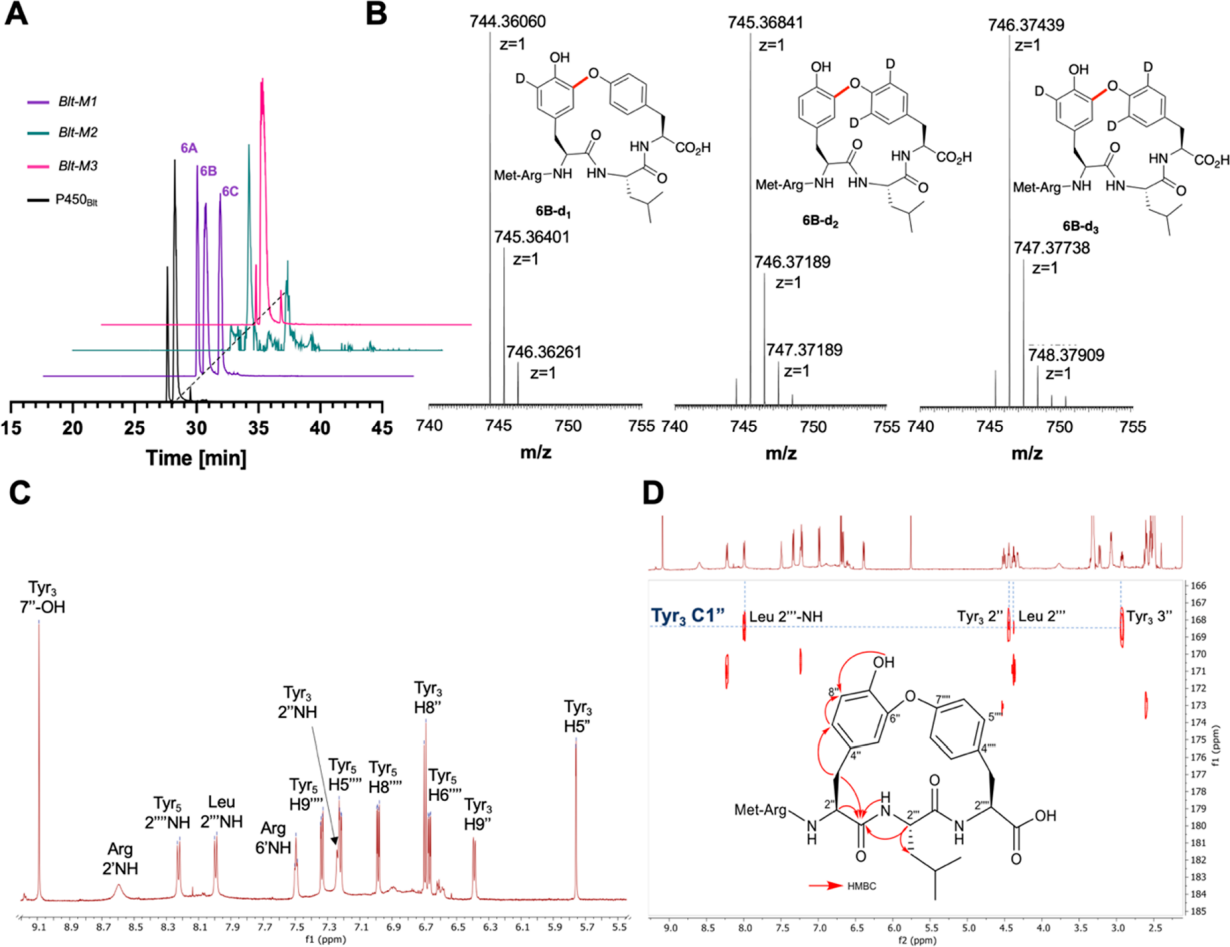
(A) Cyclization of **3** and deuterated probes **3**^**3d**^, **3**^**5d**^, and **3**^**3/5d**^ by P450_Blt_ and Blt mutants to form **6**. (B) MS^1^ isotope cluster for **6B**^**3d**^, **6B**^**5d**^, and **6B**^**3/5d**^ that agrees with the formation of a YXY cross-link via the Y-5 phenol moiety in **6B** by the *Blt-M3* mutant, as is revealed by the loss of 1 deuterium atom from **6B**^**3d**^ and **6B**^**3/5d**^ but not from **6B**^**5d**^. (C) Upfield region (extract) of the ^1^H NMR spectrum of **6B**, with peaks assigned (700 MHz, DMSO-*d*_6_). (D) Key HMBC correlations used to establish the cross-link as Tyr_3_C-Tyr_5_O in **6B** overlaid with an HMBC spectrum extract illustrating key correlations to Tyr_3_ C1″. For full correlations and spectra, see the SI.

To further characterize the specificity of these P450s for **3**, we incorporated 3,5-deuterated Tyr residues within **3** (**3**^**3d**^: *d*_2_-Tyr3; **3**^**5d**^: *d*_2_-Tyr5; **3**^**3/5d**^: *d*_2_-Tyr3-*d*_2_-Tyr5) to allow unambiguous identification of the cross-linking state by examining loss of deuterium for these probes with each P450. We validated this approach by reference to NMR analysis of **6B**, which led to the anticipated isotopic patterns from the deuterated probes **3**^**3d**^, **3**^**5d**^, and **3**^**3/5d**^ (Figure 2B). These probes then revealed that *Blt-M2* possesses a preference like *Blt-M3* for the formation of the OF-4949 type C_3_–O_5_ cross-link **6B** (>80%), albeit with much lower conversion. WT P450_Blt_ generates mostly the OF-4949 type C_3_–O_5_ cross-link **6B** (65%), with 35% forming a C_3_–C_5_ bond **6A**. This specificity was altered for *Blt-M1*, which formed all three cross-links at comparable levels (**6A**:**6B**:**6C** = 38:30:32).

To understand why the conversion of **3** by *Blt-M1* and *Blt-M3* was improved over the wildtype enzyme, we undertook MD simulations (SI Figures S27–S29). These revealed that **3** forms an H-bond network in *Blt-M1* and *Blt-M3* that is connected by the OH group of Ser239 in the I-helix and bridges the two tyrosine phenol moieties (SI Figure S27). This network is disfavored in wildtype P450_Blt_ due to the steric occlusion of the C-terminal aryl moiety by H234. However, the H234L mutation allows the C-terminal aryl moiety to pack closer to the I-helix, forming van der Waals contacts with the H234L side chain. This facilitates the conformation of the Tyr–Tyr biaryl rings seen in **6B**, whereas the presence of His234 would sterically hinder the binding of **3** in such an orientation (SI Figure S29). Interestingly, while the H234L mutation itself shows poor activity, the addition of the E238N mutation enhances activity significantly. This is likely attributable to the shorter residue enabling access to the proton relay network in this modified active site.^13^ The A231V mutation is also an important factor in determining the nature of C–O cross-linking, with MD experiments suggesting this is due to the increased steric bulk from the A231V mutation. This bulk restricts the rotameric states of sensor residue Gln-84, leading to a preference for forming a single hydrogen bond with the carbonyl of the Leu-4/Tyr-5 peptide bond. In MD simulations with wildtype P450_Blt_, Gln-84 preferentially coordinates with the amide nitrogen of the Leu-4/Tyr-5 peptide bond, whereas, in *Blt-M3*, Gln-84 can hydrogen bond to both the amide nitrogen and the carbonyl of the Leu-4/Tyr-5 peptide bond. This suggests that effective contact with the carbonyl of the Leu-4/Tyr-5 peptide bond is essential for efficient peptide conversion of **3** to form **6** (SI Figure S27).

Finally, we sought to clarify the nature of the cross-link within **5** formed by *Blt-M1* as no equivalent cross-linked tripeptide natural product has been identified. Scaled enzymatic cyclization of **Nle-2** (N-terminal Met residue was replaced with norleucine (Nle) to avoid sulfoxidation due to the release of reactive oxygen intermediates during catalysis) and purification of **Nle-5** were then performed. NMR analysis of this cross-linked peptide proved highly challenging (as has been seen for **4**)^10,13^ and although consistent with a cross-linked pentapeptide did not allow for unambiguous assignment of the location or nature of the cross-link (SI Figure S26). We turned to the use of peptide deuteration to resolve this cross-link, which based on WT biarylitide reactivity could be either C–C or C–N. This required synthesis and turnover of a deuterated version of **Nle-2** with deuterium at C_2_ of the His-3 residue (**Nle-2^3d^**).^17^ Turnover of **Nle-2**^**3d**^ by *Blt-M1* showed loss of deuterium at C-2 upon cyclization, supporting the identification of the cross-link in **Nle-5** being from the imidazole C-2 to the *m*-position of Tyr (Figure 3); no loss of deuterium was seen from remaining linear peptide due to the very slow exchange observed at this position.^17^ A control turnover of **Nle-1**^**5d**^ with P450_Blt_ also showed no loss of deuterium. The formation of a C–C cross-link by *Blt-M1* supports the role of the His234 residue in the WT enzyme in directing the formation of the C–N bond, which is supported by computational calculations.^13^

**Figure 3:**
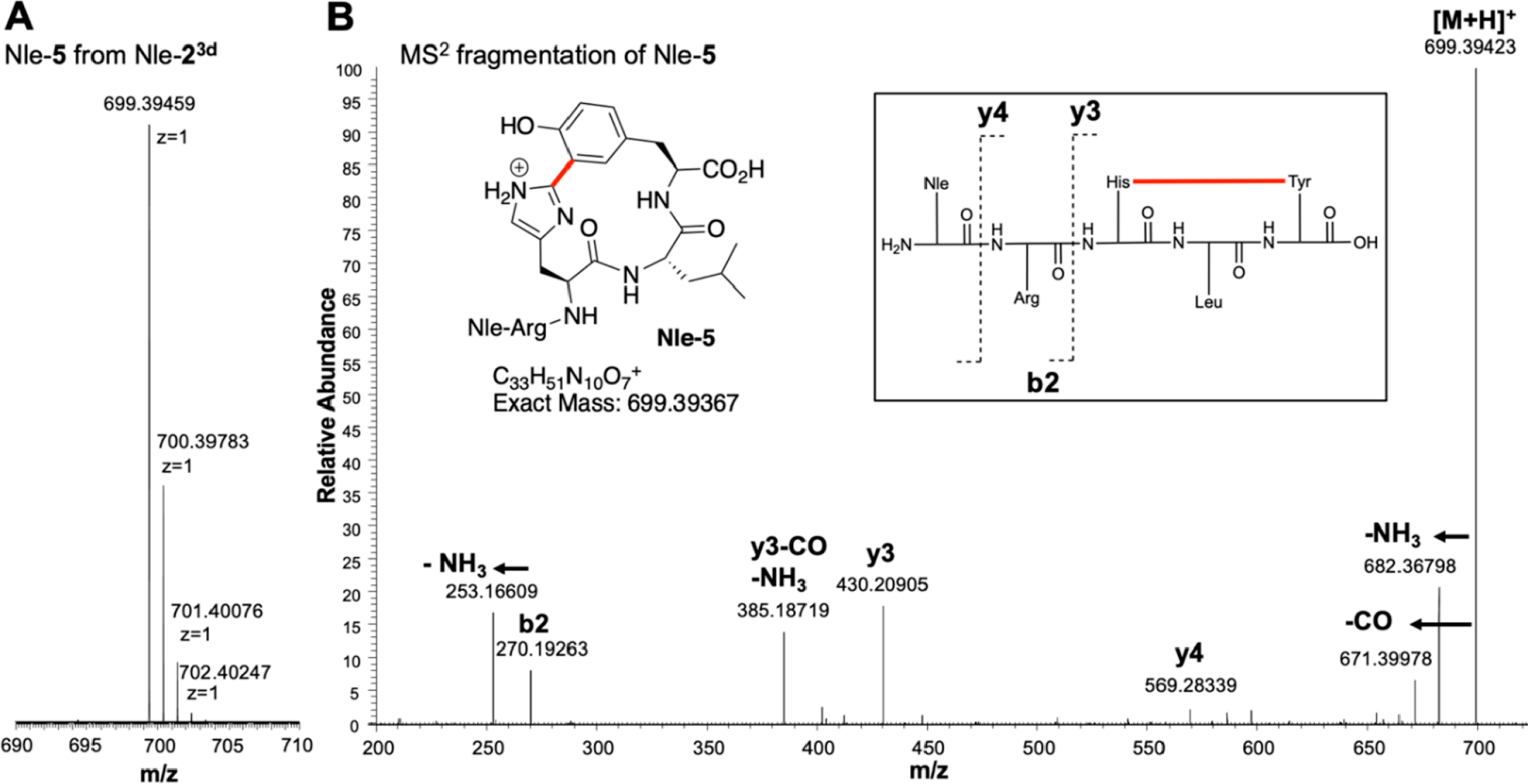
Cyclization of **Nle-2** and **Nle-2^3d^** by *Blt-M1*. (A) HRMS of the MS^1^ isotope cluster is indicative of the loss of deuterium in **Nle-5** from the cyclization of Nle-**2**^**3d**^, thus supporting a C–C cross-link in this case. (B) MS^2^ fragmentation of **Nle-5** suggesting the formation of a HXY cross-link. For NMR of **5**, see SI Figure S26.

In this study, we have shown that P450_Blt_ from biarylitide biosynthesis is highly amenable to engineering to produce further cyclic tripeptides. In addition to the Tyr-X-His and Tyr-X-Trp activity reported,^10^ here we demonstrated that mutants of P450_Blt_ can catalyze the C–C cross-linking of His-X-Tyr peptides and OF-4949 type^15^ cross-linking of Tyr-X-Tyr peptides. The potential of this enzyme to generate C–C cross-linked Tyr-X-His peptides^12^ and Tyr-X-Tyr cross-links related to K-13^16^ and arylomycin^14^ will clearly be a focus for future studies to further explore the biocatalytic potential of this potent P450.

## Data Availability

The data underlying this study are available in the published article and its Supporting Information.

## Supporting Information Available

Methods, tables, and figures including compound characterization, binding, turnover, and molecular dynamics simulations (PDF)

## References

[ref1] de la Torre B. G.; Albericio F. Peptide Therapeutics 2.0. Molecules 2020, 25, 2293 10.3390/molecules25102293. 32414106 PMC7287585

[ref2] Wenski S. L.; Thiengmag S.; Helfrich E. J. N. Complex peptide natural products: Biosynthetic principles, challenges and opportunities for pathway engineering. Synth. Systems Biotechnol. 2022, 7, 631–647. 10.1016/j.synbio.2022.01.007. PMC8842026 35224231

[ref3] Greule A.; Stok J. E.; De Voss J. J.; Cryle M. J. Unrivalled diversity: the many roles and reactions of bacterial cytochromes P450 in secondary metabolism. Nat. Prod. Rep. 2018, 35, 757–791. 10.1039/C7NP00063D. 29667657

[ref4] Aldemir H.; Shu S.; Schaefers F.; Hong H.; Richarz R.; Harteis S.; Einsiedler M.; Milzarek T. M.; Schneider S.; Gulder T. A. M. Carrier Protein-Free Enzymatic Biaryl Coupling in Arylomycin A2 Assembly and Structure of the Cytochrome P450 AryC. Chem. Eur. J. 2022, 28, e202103389 10.1002/chem.202103389. 34725865 PMC9299028

[ref5] Haslinger K.; Peschke M.; Brieke C.; Maximowitsch E.; Cryle M. J. X-domain of peptide synthetases recruits oxygenases crucial for glycopeptide biosynthesis. Nature 2015, 521, 105–109. 10.1038/nature14141. 25686610

[ref6] Tailhades J.; Zhao Y.; Ho Y. T. C.; Greule A.; Ahmed I.; Schoppet M.; Kulkarni K.; Goode R. J. A.; Schittenhelm R. B.; De Voss J. J.; Cryle M. J. A Chemoenzymatic Approach to the Synthesis of Glycopeptide Antibiotic Analogues. Angew. Chem., Int. Ed. 2020, 59, 10899–10903. 10.1002/anie.202003726. 32297389

[ref7] He B.-B.; Liu J.; Cheng Z.; Liu R.; Zhong Z.; Gao Y.; Liu H.; Song Z.-M.; Tian Y.; Li Y.-X. Bacterial Cytochrome P450 Catalyzed Post-translational Macrocyclization of Ribosomal Peptides. Angew. Chem., Int. Ed. 2023, 62, e202311533 10.1002/anie.202311533. 37767859

[ref8] Hug J. J.; Dastbaz J.; Adam S.; Revermann O.; Koehnke J.; Krug D.; Müller R. Biosynthesis of Cittilins, Unusual Ribosomally Synthesized and Post-translationally Modified Peptides from Myxococcus xanthus. ACS Chem. Biol. 2020, 15, 2221–2231. 10.1021/acschembio.0c00430. 32639716

[ref9] Nam H.; An J. S.; Lee J.; Yun Y.; Lee H.; Park H.; Jung Y.; Oh K.-B.; Oh D.-C.; Kim S. Exploring the Diverse Landscape of Biaryl-Containing Peptides Generated by Cytochrome P450 Macrocyclases. J. Am. Chem. Soc. 2023, 145, 22047–22057. 10.1021/jacs.3c07140. 37756205

[ref10] Zhao Y.; Marschall E.; Treisman M.; McKay A.; Padva L.; Crüsemann M.; Nelson D. R.; Steer D. L.; Schittenhelm R. B.; Tailhades J.; Cryle M. J. Cytochrome P450Blt Enables Versatile Peptide Cyclisation to Generate Histidine- and Tyrosine-Containing Crosslinked Tripeptide Building Blocks. Angew. Chem., Int. Ed. 2022, 61, e202204957 10.1002/anie.202204957. PMC9542247 35851739

[ref11] Hug J. J.; Frank N. A.; Walt C.; Senica P.; Panter F.; Muller R. Genome-Guided Discovery of the First Myxobacterial Biarylitide Myxarylin Reveals Distinct C-N Biaryl Crosslinking in RiPP Biosynthesis. Molecules 2021, 26, 7483 10.3390/molecules26247483. 34946566 PMC8708641

[ref12] Zdouc M. M.; Alanjary M. M.; Zarazúa G. S.; Maffioli S. I.; Crüsemann M.; Medema M. H.; Donadio S.; Sosio M. A biaryl-linked tripeptide from *Planomonospora* reveals a widespread class of minimal RiPP gene clusters. Cell Chem. Biol. 2021, 28, 733–739. 10.1016/j.chembiol.2020.11.009. 33321099

[ref13] Hansen M. H.; Keto A.; Treisman M.; Sasi V. M.; Coe L.; Zhao Y.; Padva L.; Hess C.; Leichthammer V.; Machell D. L.; Schittenhelm R. B.; Jackson C. J.; Tailhades J.; Crüsemann M.; De Voss J. J.; Krenske E. H.; Cryle M. Structural insights into a sidechain crosslinking biarylitide P450 from RiPP biosynthesis. ACS Catal. 2024, 14, 812–826. 10.1021/acscatal.3c05417.

[ref14] Höltzel A.; Schmid D. G.; Nicholson G. J.; Stevanovic S.; Schimana J.; Gebhardt K.; Fiedler H. P.; Jung G. Arylomycins A and B, new biaryl-bridged lipopeptide antibiotics produced by Streptomyces sp. Tü 6075. II. Structure elucidation. J. Antibiot. (Tokyo) 2002, 55, 571–577. 10.7164/antibiotics.55.571. 12195963

[ref15] Sano S.; Ikai K.; Katayama K.; Takesako K.; Nakamura T.; Obayashi A.; Ezure Y.; Enomoto H. OF4949, new inhibitors of aminopeptidase B. II. Elucidation of structure. J. Antibiot. 1986, 39, 1685–1696. 10.7164/antibiotics.39.1685. 3818442

[ref16] Yasuzawa T.; Shirahata K.; Sano H. K-13, a novel inhibitor of angiotensin I converting enzyme produced by Micromonospora halophytica subsp. exilisia. II. Structure determination. J. Antibiot. 1987, 40, 455–458. 10.7164/antibiotics.40.455. 3034845

[ref17] Pogostin B. H.; Malmendal A.; Londergan C. H.; Åkerfeldt K. S. pKa Determination of a Histidine Residue in a Short Peptide Using Raman Spectroscopy. Molecules 2019, 24, 405 10.3390/molecules24030405. 30678032 PMC6385126

