# Supplementary material for: An Engineered Biarylitide Cross-Linking P450 from RiPP Biosynthesis Generates Alternative Cyclic Peptides
Source: Org Lett. 2024 Feb 28;26(9):1828–33. doi: 10.1021/acs.orglett.3c04366 (PMC10929621; doi:10.1021/acs.orglett.3c04366)
Supplement: Supplementary file 1 — ol3c04366_si_001.pdf [file ol3c04366_si_001.pdf]

Supporting Information

An engineered biarylitide crosslinking P450 from RiPP biosynthesis generates alternative cyclic peptides

Maxine Treisman,[†‡¶] Laura Coe,[£¶] Yongwei Zhao,[†‡¶] Vishnu Mini Sasi,[‡£] Jemma Gullick,[†‡] Mathias H. Hansen,[†‡] Aviva Ly,[$] Victor Leichthammer,[†‡] Caroline Hess,[†‡] Daniel L. Machell,[†‡] Ralf B. Schittenhelm,[%] Joel Hooper,[$] Colin J. Jackson, [‡£^~] Julien Tailhades,[†‡] James J. De Voss,[*£] & Max J. Cryle[*†‡]

[†] Department of Biochemistry and Molecular Biology, The Monash Biomedicine Discovery Institute, Monash University; EMBL Australia; Clayton, VIC 3800, Australia

[‡] ARC Centre of Excellence for Innovations in Peptide and Protein Science, Australia

[£] School of Chemistry and Molecular Biosciences, The University of Queensland, Brisbane, QLD 4072, Australia

[#] Research School of Chemistry, The Australian National University, Acton, ACT 2601, Australia

[$] Department of Chemistry, Monash University, Clayton, VIC 3800, Australia

[%] Monash Proteomics and Metabolomics Platform, Monash University, Clayton, VIC 3800, Australia

[^] ARC Centre of Excellence in Synthetic Biology, Australian National University, Canberra, Australia

[~] Research School of Biology, Australian National University, Acton, ACT 2601, Australia

Chemicals and Reagents .... **Page 2**

Supporting Methods .... **Page 3**

Supporting Tables .... **Page 6**

Supporting Figures .... **Page 10**

Supporting References .... **Page 31**

Chemicals and Reagents

2-Chlorotrityl chloride resin (GL Biochem), DCM (Chem-supply), DMF (Ajax Finechem), methanol (Scharlau), TFA (Oakwood chemical), TIPS (Sigma-Aldrich), diethyl ether (Sigma-Aldrich), OxymaPure (Novabiochem), DIC (Sigma-Aldrich), KH_2PO_4 (Sigma-Aldrich), K_2HPO_4 (Sigma-Aldrich), tryptone (MP Biochemicals), yeast extract (MP Biochemicals), SIGMAFAST protease inhibitor cocktail tablets (EDTA-free ; Sigma-Aldrich), FA (Sigma-Aldrich), HEPES (Sigma-Aldrich), IPTG (Promega), Imidazole (Sigma-Aldrich), Tris-HCl (Sigma-Aldrich), δ-aminolevulinic acid (Sigma-Aldrich), NaCl (Sigma-Aldrich), $MgCl_2$ (Sigma-Aldrich), glucose (Sigma-Aldrich), glucose dehydrogenase (Sigma-Aldrich), NADH (Sigma-Aldrich), Kanamycin sulfate (Sigma-Aldrich), Gentamycin sulfate (Sigma-Aldrich), 5-alpha competent *E. coli* cells (NEB), ArcticExpress (DE3) competent cells (Agilent), Ni-NTA agarose (Macherey-Nagel), Precision Plus Protein^{TM} Dual Colour Standards (Bio-Rad), SPE Bond elute (Agilent), C18 Strata-X-SPE-cartridges (Phenomenex).

Supporting Methods

Deuterated Amino Acid Synthesis

*L-tyrosine-(*phenyl-*3,5-d2)*

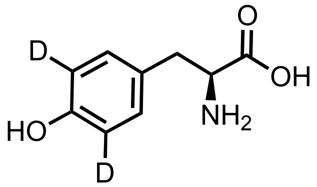

To a solution of L-tyrosine (250 mg, 1.38 mmol) in D_2O (10 mL) was added DCl (35% in D_2O, 1 mL). The mixture was stirred and heated to reflux overnight with an oil-bath temperature of 120°C. The heat was lowered to 40 °C and the solvent evaporated under a stream of nitrogen to afford the crude product as a yellow solid (99% deuteration by ^{1}H NMR). ^{1}H-NMR (400 MHz, D_2O) δ 6.32 (2H, s), 6.02 (0.02H, d), 3.47 (1H, dd, *J* = 7.4, 5.6 Hz), 2.40 (1H, dd, *J* = 14.7, 5.6 Hz), 2.29 (1H, m, *J* = 14.7, 7.4 Hz).

*Fmoc-L-tyrosine-(*phenyl-*3,5-d_2)*

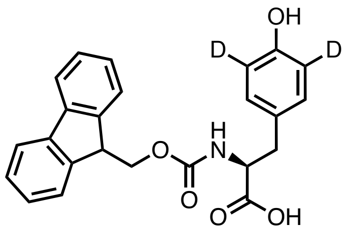

L-tyrosine-(phenyl-3,5-d2) (253 mg, 1.38 mmol) was dissolved in a solution of THF (2.05 mL), water (2.05 mL) and sodium bicarbonate (348 mg, 4.14 mmol) at room temperature. Fmoc-succinimide (558.6 mg, 1.66 mmol) was added to the solution and stirred at room temperature overnight. The solvent was removed under reduced pressure. 10% HCl was added to ensure a pH of 3, and an aqueous workup was performed with water and EtOAc. Flash chromatography (30-100% EtOAc/hexanes) afforded the pure product as light-yellow crystals (297 mg, 53% yield). ^{1}H-NMR (400 MHz, MeOD) δ 7.79 (2H, d, *J* = 7.1 Hz), 7.60 (2H, d, *J* = 7.6 Hz), 7.43-7.35 (2H, m), 7.34-7.26 (2H, m), 7.06 (2H, s), 4.37-4.28 (2H, m), 4.27-4.15 (2H, m), 3.11 (1H, d, *J* =14.3 Hz), 2.85 (1H, t, *J* = 11.7 Hz). ^{13}C-NMR (100 MHz, MeOD) δ 173.02, 158.36, 157.16, 145.25, 145.18, 142.53, 131.21, 129.27, 128.75, 128.17, 126.33, 126.24, 121.08, 120.87, 115.96. R_f: 0.21 (100% EtOAc). MS: $[M+H]^+$ calculated *m/z* = 406.1624, found *m/z* = 406.1616.

Supporting Methods

Peptide Synthesis

Peptide synthesis of **1-3** was performed as automatically as previously described, using a Liberty Blue Automated Microwave Peptide Synthesizer (CEM) at a 0.1 mmol scale.[1] Synthesis of Nle-1^{5d} (Nle-RYL-His(C2-d) and Nle-2^{3d} (Nle-R-His(C2-d)-LY) was performed as above. The peptide then underwent deuterium exchange by incubating in ~10 mM D_2O at pH 8 at 50 ºC for 72 hours according to a previously reported protocol.[2]

Plasmid Construction

The $P450_{Blt}$ gene was synthesized with codon optimization for *E. coli* and ligated into a pET28a(+) vector (Twist Bioscience) following a previously reported protocol.[1]

Generation of P450 mutants

Site-directed mutagenesis was performed to generate several $P450_{Blt}$ mutants according to an optimized Quikchange™ Site-Directed Mutagenesis (Agilent Technologies) protocol. To generate the mutants, pairs of primers were designed, and the vector amplified by PCR (**SI Table S1**). High fidelity PCR was carried out with 12.5 µL Phusion® High Fidelity DNA Polymerase MasterMix (NEB), 1 µL of 10 µM $P450_{Blt}$ plasmid as the template, 1.25 µL of 10 µM forward and reverse primers, and 0.75 µL of DMSO and nuclease-free water to make up a 25 µL reaction. The PCR reaction was then placed in a T100 thermocycler (Bio-Rad). An initial denaturation step (30 seconds, 98°C) was then followed by 32 cycles of denaturation (10 seconds, 98°C), annealing (30 seconds, 60°C) and elongation (30 seconds/1000 bps, 72°C). After completion of the cycles a final elongation step was performed (2 minutes, 72°C). Parental template DNA was digested using DpnI restriction endonuclease (NEB). The PCR products were transformed into DH5α competent *E. coli* (as described in [1]). Clones were selected for using 1% LB agar plates supplemented with kanamycin. Plasmids were isolated and purified using the GeneJET Plasmid Miniprep kit and protocol. The desired mutations were verified by Sanger sequencing (Garvan Institute).

Protein Expression and Purification

Test expressions as well as large scale expression and 3-step purification (Ni-NTA affinity, anion exchange, gel filtration) of P450 mutant enzymes were performed as has been previously reported.[1, 3]

UV-Vis Spectroscopy

The UV-visible spectra of the P450 enzymes were obtained using a Jasco V-750 spectrophotometer as has been previously reported.[1] The spectra were measured until it reached a stable peak. CO difference spectra were obtained using a Jasco V-750 spectrophotometer at 30 °C as has been previously described.[1] The UV-vis spectra were measured between 390–600 nm every 5 mins until the spectra was stable.[1, 4] Substrate interaction with the P450s was measured using UV-visible absorbance spectroscopy carried out on a Jasco V-750 spectrophotometer according to previously reported protocol.[1] Amplitudes of the different spectra were extrapolated ($\Delta A = Amax - Amin$) and plotted against the substrate concentration. Curve fitting of the resulting data points was performed using GraphPad Prism 9 by applying a one-site binding model: $Y=Bmax*X/(KD+X) + NS*X + Background$ (Y = delta Abs, Bmax = the maximum specific binding, KD = equilibrium dissociation constant, NS = slope of nonspecific binding, Background = nonspecific binding

with no added ligand).[1] Each enzyme-substrate pair was assayed in triplicate and the average K_d determined.

P450-Mediated Cyclization Reactions

Peptide cyclisation was performed using the P450 enzyme as well as redox partners as has been previously reported.[1] Peptides were synthesized using reported protocols.[5, 6] The reaction was purified using solid-phase extraction column (Bond elute, Agilent) as performed previously. Following concentration, the samples were analyzed on LCMS using an Agilent ZORBAX 300SB-C18 5 µm column as previously reported.[1] Each reaction was carried out in triplicate and data was analyzed using Excel and GraphPad Prism 9. Conversions are calculated based on the area under curve of EICs (extracted ion chromatograms). Large-scale enzymatic cyclisation was carried out to obtain sufficient material for analysis by NMR as has been previously reported.[1]

LCMS and HRMS Analysis

Peptide analysis was conducted on a Shimadzu HPLC-MS system (LCMS-2020 with a Phenomenex Zorbax 300SB-C18 column, ESI operating in positive and negative mode; or LCMS-8050 with a Phenomenex Aeris 1.7 µm PEPTIDE XB-C18 100 column, ESI operating in positive and negative mode). The mobile phases used were water + 0.1% FA and ACN + 0.1% FA for analytical runs.

High-resolution mass spectrometry measurements were performed on an Orbitrap Fusion mass spectrometer (Thermo Scientific) coupled to a Dionex UltiMate 3000 RSLCnano system equipped with a Dionex UltiMate 3000 RS autosampler, an Acclaim PepMap RSLC analytical column (75 µm x 50 cm, nanoViper, C18, 2 µm, 100Å; Thermo Scientific) and an Acclaim PepMap 100 trap column (100 µm x 2 cm, nanoViper, C18, 5 µm, 100Å; Thermo Scientific) as previously reported.[1]

$P450_{Blt}$: Linear **Nle-1**^{5d} HRMS (ESI) m/z: $[M + H]^+$ Calcd for $C_{33}H_{52}DN_{10}O_7^+$ 702.41560; Found 702.41577. Cyclic **Nle-1**^{5d} HRMS (ESI) m/z: $[M + H]^+$ Calcd for $C_{33}H_{50}DN_{10}O_7^+$ 700.39995; Found 700.39899.

Blt-M1: Linear **1** HRMS (ESI) m/z: $[M + H]^+$ Calcd for $C_{32}H_{51}N_{10}O_7S^+$ 719.36574; Found 719.36536. Cyclic **1** HRMS (ESI) m/z: $[M + H]^+$ Calcd for $C_{32}H_{49}N_{10}O_7S^+$ 717.35009; Found 717.39412 (rt 22.04), 717.34894 (rt 23.78). Linear **2** HRMS (ESI) m/z: $[M + H]^+$ Calcd for $C_{32}H_{51}N_{10}O_7S^+$ 719.36574; Found 719.36554. Cyclic **2** HRMS (ESI) m/z: $[M + H]^+$ Calcd for $C_{32}H_{49}N_{10}O_7S^+$ 717.35009; Found 717.34979 (rt 23.14), 717.34973 (rt 24.49). Linear **3** HRMS (ESI) m/z: $[M + H]^+$ Calcd for $C_{35}H_{53}N_8O_8S^+$ 745.37016; Found 745.36945. Cyclic **3** HRMS (ESI) m/z: $[M + H]^+$ Calcd for $C_{35}H_{51}N8O_8S^+$ 743.35451; Found 743.35400 (rt 28.64), 743.35394 (rt 29.29), 743.35394 (rt 30.62). Linear **Nle-2**^{3d} HRMS (ESI) m/z: $[M + H]^+$ Calcd for $C_{33}H_{52}DN_{10}O_7^+$ 702.41560; Found 702.41687. Cyclic **Nle-2**^{3d} HRMS (ESI) m/z: $[M + H]^+$ Calcd for $C_{33}H_{51}N_{10}O_7^+$ 699.39367; Found 699.39459.

Blt-M2: Linear **1** HRMS (ESI) m/z: $[M + H]^+$ Calcd for $C_{32}H_{51}N_{10}O_7S^+$ 719.36574; Found 719.36755. Linear **2** HRMS (ESI) m/z: $[M + H]^+$ Calcd for $C_{32}H_{51}N_{10}O_7S^+$ 719.36574; Found 719.36737. Linear **3** HRMS (ESI) m/z: $[M + H]^+$ Calcd for $C_{35}H_{53}N_8O_8S^+$ 745.37016; Found 745.37128. Cyclic **3** HRMS (ESI) m/z: $[M + H]^+$ Calcd for $C_{35}H_{51}N8O_8S^+$ 743.35451; Found 743.35583 (rt 27.86), 743.35583 (rt 29.31), 743.35510 (rt 30.95).

Blt-M3: Linear **1** HRMS (ESI) m/z: $[M + H]^+$ Calcd for $C_{32}H_{51}N_{10}O_7S^+$ 719.36574; Found 719.36493. Cyclic **1** HRMS (ESI) m/z: $[M + H]^+$ Calcd for $C_{32}H_{49}N_{10}O_7S^+$ 717.35009; Found

717.34955. Linear **2** HRMS (ESI) m/z: $[M + H]^+$ Calcd for $C_{32}H_{51}N_{10}O_7S^+$ 719.36574; Found 719.36536. Cyclic **2** HRMS (ESI) m/z: $[M + H]^+$ Calcd for $C_{32}H_{49}N_{10}O_7S^+$ 717.35009; Found 717.34906 (rt 22.84), 717.34979 (rt 24.33). Linear **3** HRMS (ESI) m/z: $[M + H]^+$ Calcd for $C_{35}H_{53}N_8O_8S^+$ 745.37016; Found 745.36938. Cyclic **3** HRMS (ESI) m/z: $[M + H]^+$ Calcd for $C_{35}H_{51}N8O_8S^+$ 743.35451; Found 743.35394 (rt 29.01), 743.35394 (rt 29.26), 743.35400 (rt 31.04).

NMR Analysis

Nuclear magnetic resonance (NMR) spectra of deuterated building blocks and linear peptides were collected using a Bruker Avance III NMR spectrometer equipped with a 9.4 T magnet and 5 mm BBFO probe, operating at 400 MHz (^1H), 101 MHz (^{13}C).

Nuclear magnetic resonance (NMR) spectra of crosslinked peptides were collected using a Bruker Avance III HD equipped with a 16.4 T magnet and TCI cryoprobe operating at 700 MHz (^1H), 176 MHz (^{13}C), 71 MHz (^{15}N).

Chemical shifts (δ) are reported in parts per million (ppm) and referenced to the residual solvent signals. ^{15}N spectra are referenced to liquid ammonia δ_N 0 ppm using the Bruker program Xiref. Coupling constants, *J* are reported in Hz.

Molecular Dynamics Simulations

$P450_{Blt}$ mutants and alternative substrate complexes were prepared as described below. The Glide module from Schrödinger[7] was used to perform docking studies with substrate analogues of MRYLH. **2** and **3** were generated using the Build module in Maestro,[8] with the conformation of MRYLH as the template, and were prepared using the LigPrep program.[9] *Blt-M1* and *Blt-M3* models were generated from the crystal structure of the wild type $P450_{Blt}$ (PDB 8U2M) using the Residue Scanning Calculations module from BioLuminate,[10] with backbone minimization set to 5 Å around the selected residue. The Receptor Grid Generation module from Glide was used to define the docking grid box, and Standard Precision was set with a rigid docking protocol adapted for final refinement, generating a maximum of 10 poses for each ligand. The top scoring ligand pose was used in following molecular dynamic simulations. Molecular modelling studies and dynamic simulations were carried out as previously described.[1] 200 ns simulations were run in triplicate after an initial 1 ns minimisation utilising the Schrodinger Molecular Dynamics module.

Supporting Tables

Supporting Table S1. Primer sequences used for mutant generation.

Description	Template	#	Sequence
$P450_{Mut1}$	WT $P450_{Blt}$	fwd	CAACTCGCTGCGTGTTGTGCTGCTCGCTG
	WT $P450_{Blt}$	rev	CAGCGAGCAGCACAACACGCAGCGAGTTG
$P450_{Mut2}$	$P450_{Mut1}$	fwd	GTTACAACAGTGTGTCCCGTC
	$P450_{Mut1}$	rev	GACACACTGTTGTAACCAGCG
$P450_{Mut3}$	WT $P450_{Blt}$	fwd	CGTGCTGTGCTGCTCGCTGGTTACAATAGTGTGTCCCGTC
	WT $P450_{Blt}$	rev	GACGGGACACACTATTGTAACCAGCGAGCAGCACAGCACG

# - indicates the directionality of the primer

Supporting Table S2. Summary of HRMS and MS^2 data for peptide species generated in P450$_{Blt}$ mutant turnovers.

Enzyme	Substrate entry		m/z (detected, peak apex)	RT (min, peak apex)	Putative composition	Theoretical m/z	Mass error (ppm)	MSMS isolation (m/z)	MSMS spectral RT	Putative sequence	Notes (main sequence ions detected with losses as * = -NH3, ^ = -H2O, + = +H2O)
P450$_{Blt}$	Nle-1[5d]	Linear	702.41577	22.99	1Nle1Arg1Tyr1Leu1His	702.41560	0.24	702.41553	23.00	Nle-RYLH	b2*, b2, b3*, y3, b3+, a4, b4, b4+, y4*, y4, M*
		Cyclic	700.39899	21.04	1Nle1Arg1Tyr1Leu1His	700.39995	-1.37	700.39874	21.05	Nle-RYLH	b2, y4, z4, y3, M*
Blt-M1 (A252V/ H255L)	1	Linear	719.36536	25.12	1Met1Arg1Tyr1Leu1His	719.36574	-0.53	719.36475	25.13	MRYLH	y1, a2*, y2, b2*, b2, x2, c2, a3*, a3, y3, b3*, b3+, a4*, a4, b4*, b4, y4*, b4+, y4, M*
		Cyclic	717.34912	22.04	1Met1Arg1Tyr1Leu1His	717.35009	-1.35	717.34924	22.03	MRYLH	x4, y4, z4, y3, c2, M*
			717.34894	23.78			-1.60	717.34955	23.79	MRYLH	a1, z4, b2, y3, M*
Blt-M1 (A252V/ H255L)	2	Linear	719.36554	25.27	1Met1Arg1His1Leu1Tyr	719.36574	-0.28	719.36499	25.23	MRHLY	a2*, b2*, b2, y2, c2, a3*, a3, b3*, y3, c3, b3+, a4*, a4, b4*, b4, b4+, y4*, y4, M*
		Cyclic	717.34979	23.14	1Met1Arg1His1Leu1Tyr	717.35009	-0.42	717.34906	23.15	MRHLY	z4, b2, y3, c2, M*
			717.34973	24.49			-0.50	717.34924	24.53	MRHLY	a1, z4, x3, b2, y3, c2, z3, M*
Blt-M1 (A252V/ H255L)	3	Linear	745.36945	31.84	1Met1Arg2Tyr1Leu	745.37016	-0.95	745.36957	31.85	MRYLY	a2*, b2*, b2, y2, a3*, a3, b3*, b3, y3, b3+, a4*, a4, b4*, b4, b4+, z4, y4
		Cyclic	743.35400	28.64	1Met1Arg2Tyr1Leu	743.35451	-0.69	743.35394	28.64	MRYLY	a1, y4, c1, z4, x3, b2, y3, z3, M*
			743.35394	29.29			-0.77	743.35382	29.35	MRYLY	a1, y4, c1, z4, a2, x3, b2, y3, c2, z3, M*
			743.35394	30.62			-0.77	743.35364	30.65	MRYLY	a1, y4, c1, z4, x3, b2, y3, c3, z3, M*
Blt-M1 (A252V/ H255L)	Nle-2[3d]	Linear	702.41687	24.02	1Nle1Arg1His1Leu1Tyr	702.4156	1.81	702.41589	24.01	NleRHLY	b2*, b2, y2, b3*, y3, a4, b4*, b4, b4+, y4*, M*
		Cyclic	699.39459	21.88	1Nle1Arg1His1Leu1Tyr	699.39367	1.32	699.39423	21.88	NleRHLY	a1, z4, x3, b2, y3, c2, z3, M*
Blt-M2 (A252V/ E259N)	1	Linear	719.36755	26.76	1Met1Arg1Tyr1Leu1His	719.36574	2.52	719.36719	26.81	MRYLH	y1, b2*, b2, b3*, b3+, a4, b4, b4+, M*
Blt-M2 (A252V/ E259N)	2	Linear	719.36737	29.62	1Met1Arg1His1Leu1Tyr	719.36574	2.27	719.36639	29.59	MRHLY	b2*, c2, b3*, y3, b3+, a4*, a4, b4*, b4, b4+, y4*, M*
Blt-M2 (A252V/ E259N)	3	Linear	745.37128	31.80	1Met1Arg2Tyr1Leu	745.37016	1.50	745.37024	31.80	MRYLY	y1, b2*, b2, y2, a3*, b3*, b3, a4, b4, b4+, y4*, M*
		Cyclic	743.35583	27.86	1Met1Arg2Tyr1Leu	743.35451	1.78	743.35522	27.85	MRYLY	a1, y4, z4, x3, b2, y3, z3, M*
			743.35583	29.31		743.35451	1.78	743.35516	29.27		a1, y4, c1, z4, x3, b2, y3, c2, z3, M*
			743.35510	30.95		743.35451	0.79	743.35529	30.90		a1, y4, z4, b2, y3, M*
Blt-M3 (H255L/ E259N)	1	Linear	719.36493	24.41	1Met1Arg1Tyr1Leu1His	719.36574	-1.13	719.36505	24.46	MRYLH	y1, a2*, z2, y2, b2*, b2, x2, a3*, a3, y3, b3*, b3, b3+, a4*, a4, b4*, b4, y4*, b4+, y4, M*
		Cyclic	717.34955	23.63	1Met1Arg1Tyr1Leu1His	717.35009	-0.75	717.34912	23.64	MRYLH	x4, y4, z4, x3, b2, y3, z3, M*
Blt-M3 (H255L/ E259N)	2	Linear	719.36536	24.84	1Met1Arg1His1Leu1Tyr	719.36574	-0.53	719.36487	24.85	MRHLY	a2, b2*, b2, y2, c2, a3*, a3, b3*, b3, y3, c3, b3+, x3, a4*, a4, b4+, b4, b4+, y4*, y4, M*
		Cyclic	717.34906	22.84	1Met1Arg1His1Leu1Tyr	717.35009	-1.44	717.34955	22.85	MRHLY	a1, z4, b2, y3, M*
			717.34979	24.33	1Met1Arg1His1Leu1Tyr	717.35009	-0.42	717.34955	24.36	MRHLY	a1, z4, x3, b2, y3, c2, z3, M*
Blt-M3 (H255L/ E259N)	3	Linear	745.36938	32.06	1Met1Arg2Tyr1Leu	745.37016	-1.05	745.36926	32.06	MRYLY	c1, y1, b2*, b2, y2, a3*, a3, b3*, b3, y3, c3, b3+, a4*, a4, b4*, b4, b4+, y4*, y4, M*
		Cyclic	743.35394	29.01	1Met1Arg2Tyr1Leu	743.35451	-0.77	743.35376	29.01	MRYLY	a1, y4, c1, z4, a2, x3, b2, y3, z3, M*
			743.35394	29.26			-0.77	743.35394	29.27	MRYLY	a1, y4, c1, z4, b2, y3, c2, z3, M*
			743.35400	31.04			-0.69	743.35382	31.01	MRYLY	a1, y4, c1, z4, x3, b2, y3, c2, z3, M*

Supporting Table S3. Summary of NMR data used to establish the structure of **6B**.

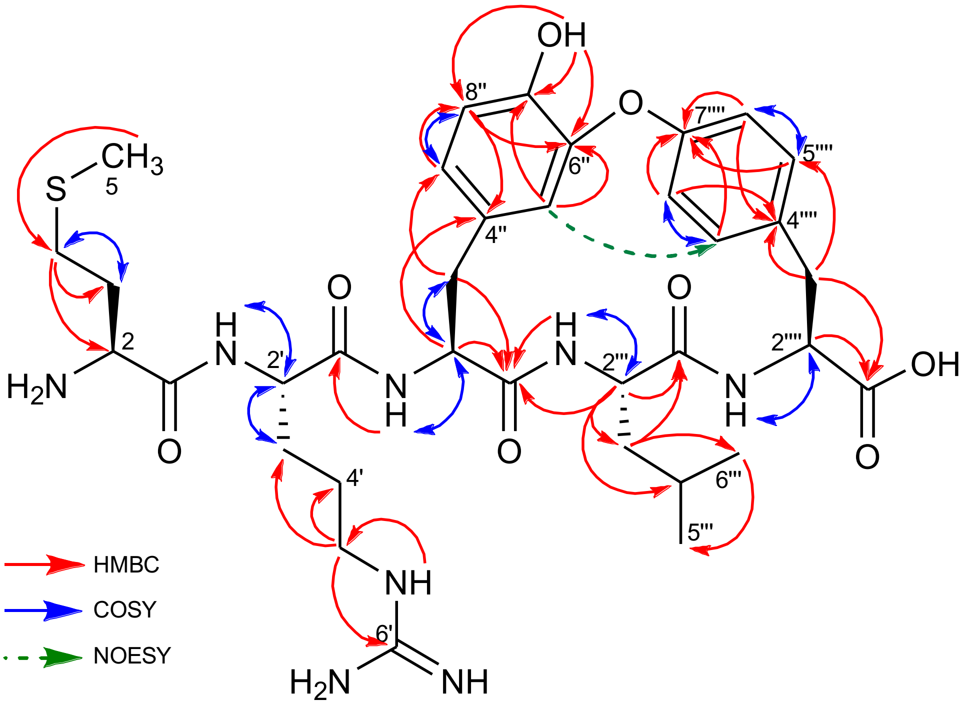

Key HMBC, COSY and NOESY correlations used to establish the structure of **6B**.

^{1}H, ^{13}C and ^{15}N NMR assignments for crosslinked MRYLY (**6**) 700 MHz, DMSO-d$_6$			
	**δ_H (*J*)**	**δ_C**	**δ_N[b]**
**1**	-	Not obs.	
**2**	3.76 (s, br)	51.9[a]	
**3**	1.85-1.92 (m, br), 1.96-2.03 (m, br)	31.6[a]	
**4**	2.54 (t, 7.0, ov)	28.5	
**5**	2.11 (s)	14.4	
**1'**	-	170.4	
**2'**	4.33 (dd, 13.6, 6.8)	52.6	
**2'-NH**	8.59 (br)	-	Not obs.
**3'**	1.44-1.51 (m, ov), 1.62-1.67 (m)	29.1	
**4'**	1.48-1.54 (m, ov), 1.40-1.47 (m, ov)	25.2	
**5'**	3.07 (ddd, 6.0, 6.9)	40.4	
**6'**	-	156.8[a]	
**6'-NH**	7.50 (t, 6.0)	-	84.9
**1''**	-	168.3[a]	
**2''**	4.45 (td, 6.5, 1.6, br)	51.2	
**2''-NH**	7.23 (d, 6.5, ov)	-	113.1
**3''**	2.93 (dd, 13.9, 6.5), 2.57-2.60 (m, ov)	36.5	

**4”**	-	126.7[a]	
**5”**	5.76 (d, 2.0)	115.9	
**6”**	-	147.4[a]	
**7”**	-	145.0[a]	
**7”-OH**	9.09 (s)	-	
**8”**	6.70 (d, 8.2)	115.7	
**9”**	6.39 (dd, 8.2, 2.0)	123.9	
**1”’**	-	171.0[a]	
**2”’**	4.38 (dt, 9.6, 4.5)	49.6	
**2”’-NH**	8.00 (d, 9.6)	-	117.4
**3”’**	1.36-1.44 (m, ov)	42.6	
**4”’**	1.45-1.52 (m, ov)	24.1	
**5”’**	0.84 (d, 6.5, ov)	21.8	
**6”’**	0.83 (d, 6.5, ov)	23.5	
**1””**	-	173.0[a]	
**2””**	4.52 (ddd, 12.9, 10.0, 3.6)	52.9	
**2””-NH**	8.22 (d, 10.0)	-	114.1
**3””**	3.23 (dd, 12.9, 3.6), 2.61 (ov)	37.1	
**4””**	-	134.1[a]	
**5””**	7.22 (dd, 8.3, 2.2)	131.7	
**6””**	6.67 (dd, 8.3 2.6)	120.6	
**7””**	-	153.4[a]	
**8””**	6.99 (dd, 8.3, 2.6)	121.5	
**9””**	7.34 (dd, 8.3, 2.2)	130.3	

s = singlet, d = doublet, t = triplet, m = multiplet, dd = doublet of doublets, ddd = doublet of doublet of doublets, dt = doublet of triplets, br = broad signal, ov = overlapped signal. Not Obs. = signal not observed.
[a] Chemical shift assignments from ^{1}H-^{13}C HMBC spectra.
[b] Chemical shift assignments from ^{1}H-^{15}N HSQC spectra.

Supporting Figures

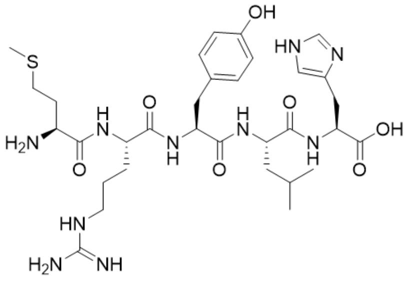

Chemical Formula: $C_{32}H_{50}N_{10}O_7S$
Exact Mass: 718.35846
Molecular Weight: 718.87500

1

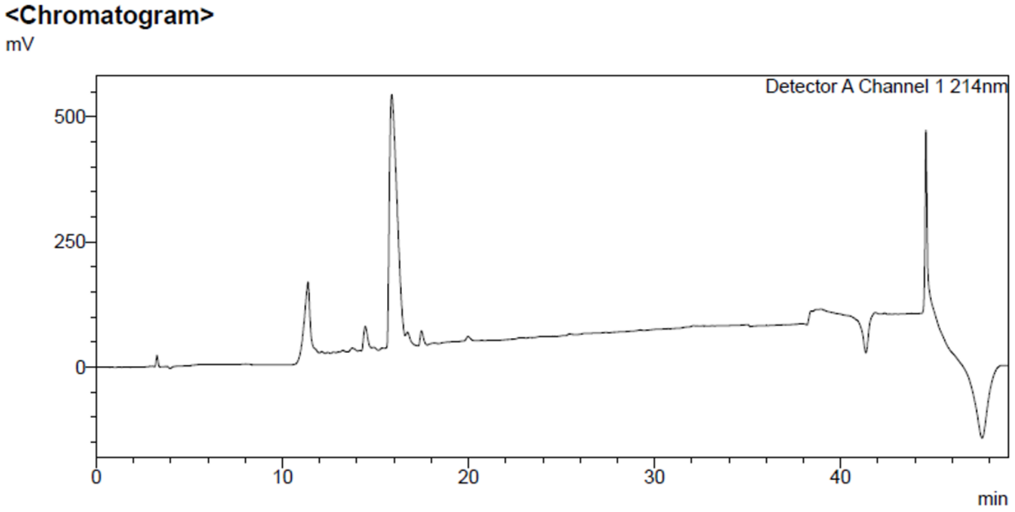

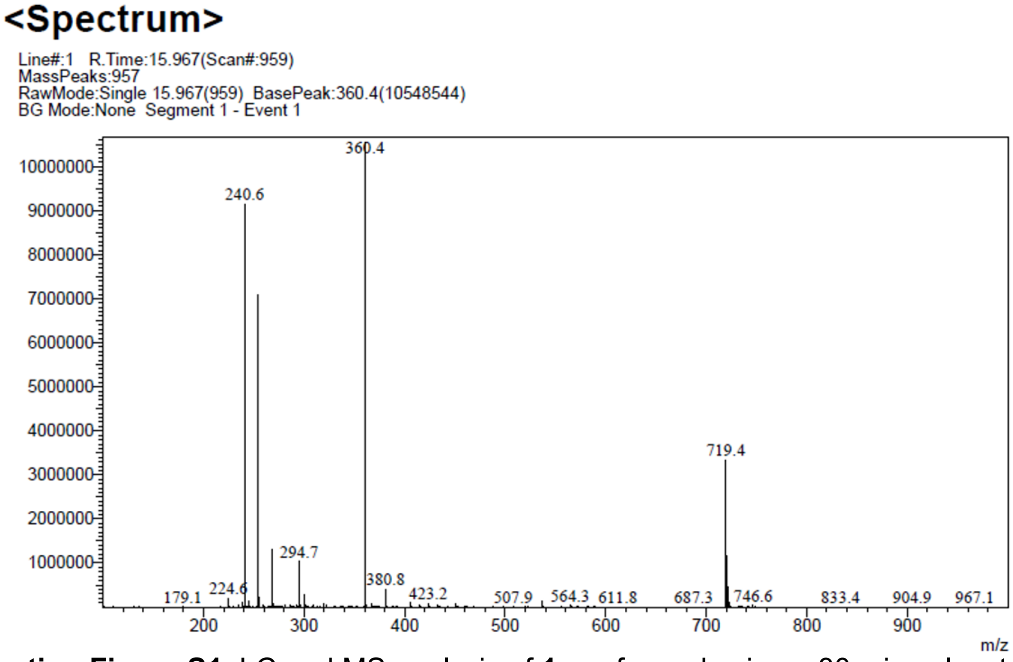

Supporting Figure S1. LC and MS analysis of **1**, performed using a 30 min solvent gradient from 0% to 50% (5-35 min) ACN in water (+ 0.1% FA) with a flow rate of 1 mL/min.

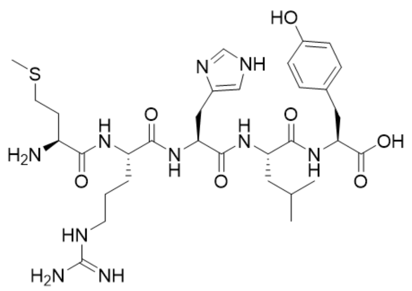

Chemical Formula: $C_{32}H_{50}N_{10}O_7S$
Exact Mass: 718.35846
Molecular Weight: 718.87500

2

<Chromatogram>

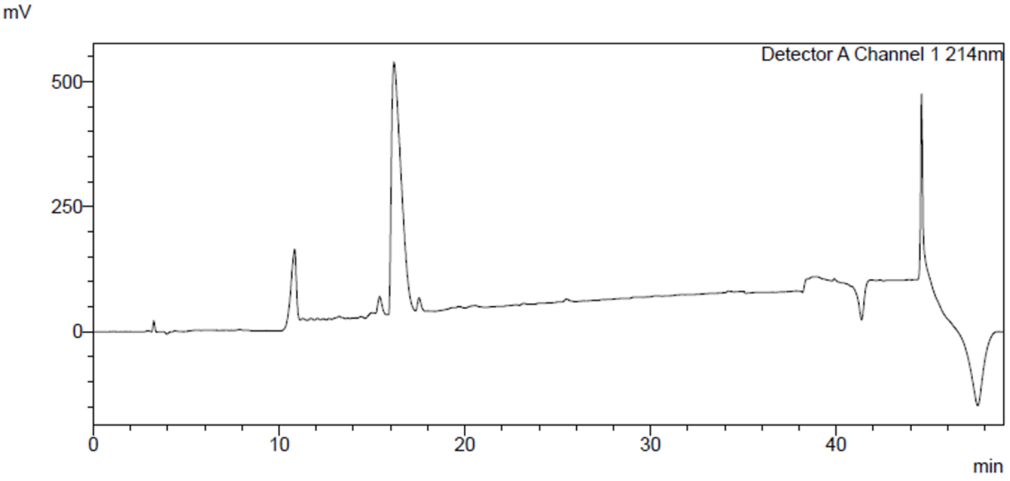

<Spectrum>

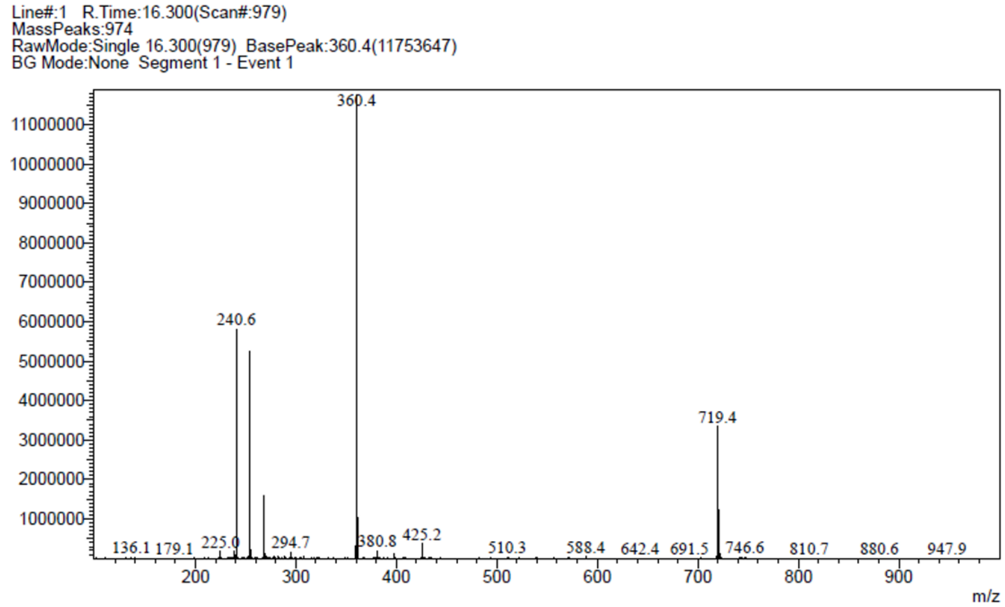

Supporting Figure S2. LC and MS analysis of **2**, performed using a 30 min solvent gradient from 0% to 50% (5-35 min) ACN in water (+ 0.1% FA) with a flow rate of 1 mL/min.

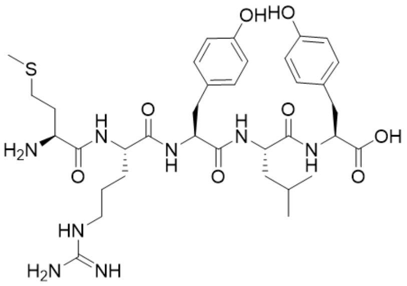

Chemical Formula: $C_{35}H_{52}N_8O_8S$
Exact Mass: 744.36288
Molecular Weight: 744.90900

3

<Chromatogram>

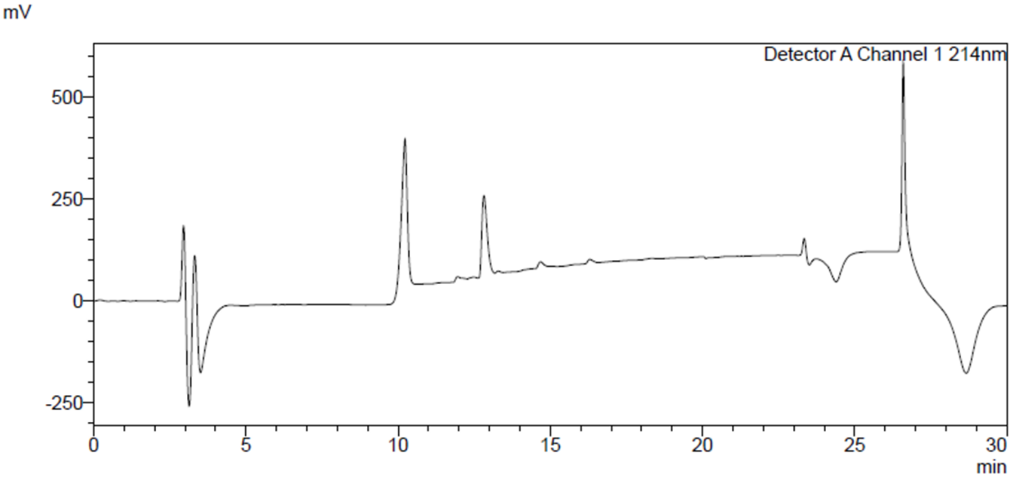

<Spectrum>

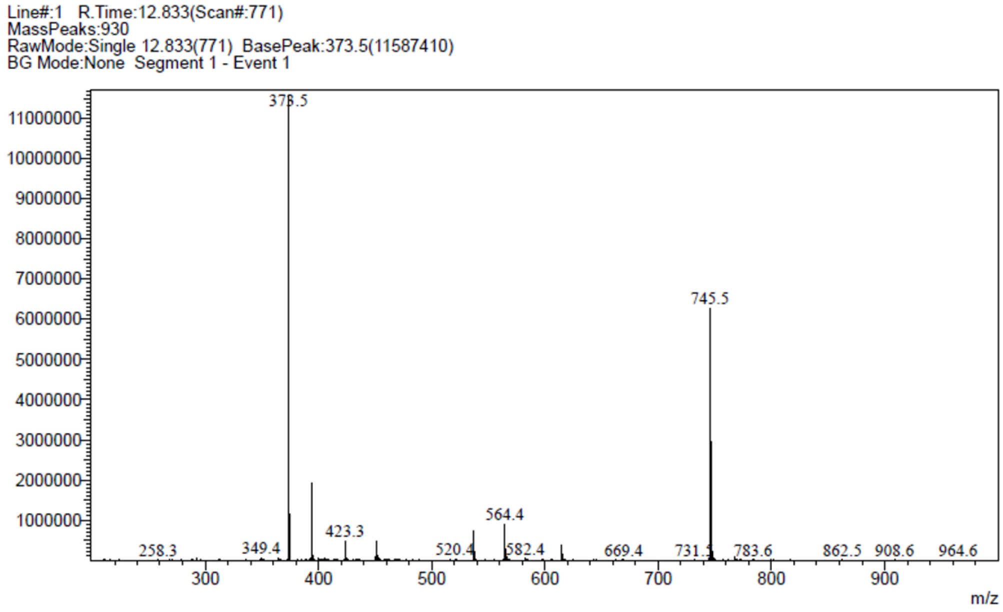

Supporting Figure S3. LC and MS analysis of **3**, performed using a 30 min solvent gradient from 0% to 90% (5-25 min) ACN in water (+ 0.1% FA) with a flow rate of 1 mL/min.

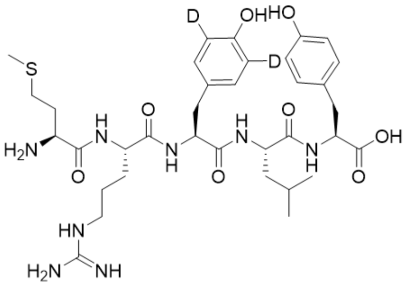

Chemical Formula: $C_{35}H_{50}D_2N_8O_8S$
Exact Mass: 746.37543
Molecular Weight: 746.92120

3^{3d}

<Chromatogram>

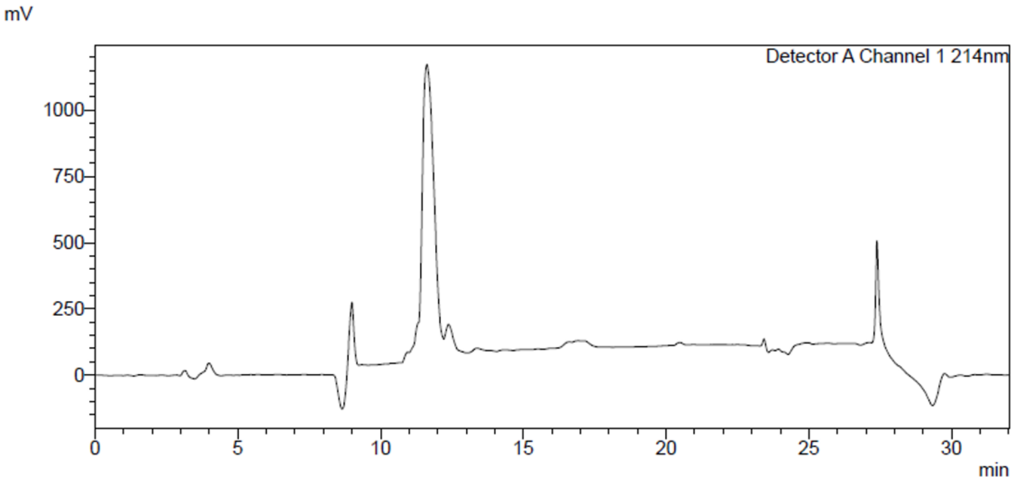

<Spectrum>

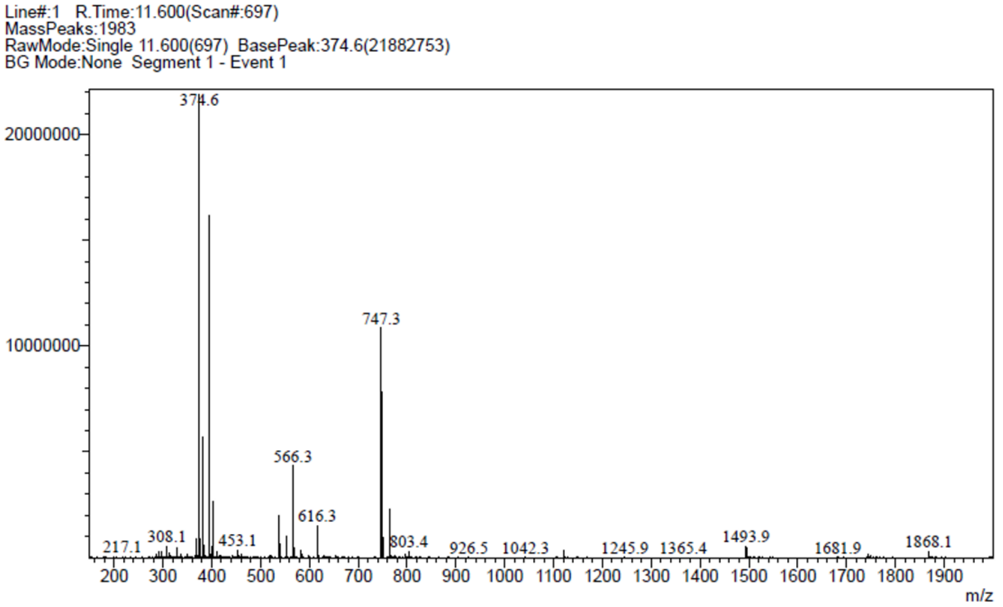

Supporting Figure S4. LC and MS analysis of **3^{3d}**, performed using a 30 min solvent gradient from 0% to 95% (5-25 min) ACN in water (+ 0.1% FA) with a flow rate of 1 mL/min.

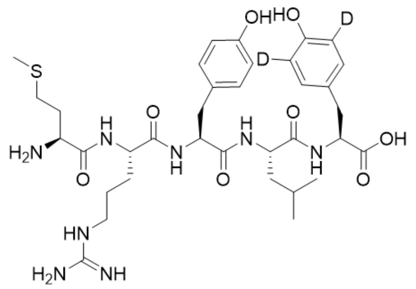

Chemical Formula: $C_{35}H_{50}D_2N_8O_8S$
Exact Mass: 746.37543
Molecular Weight: 746.92120

3^{5d}

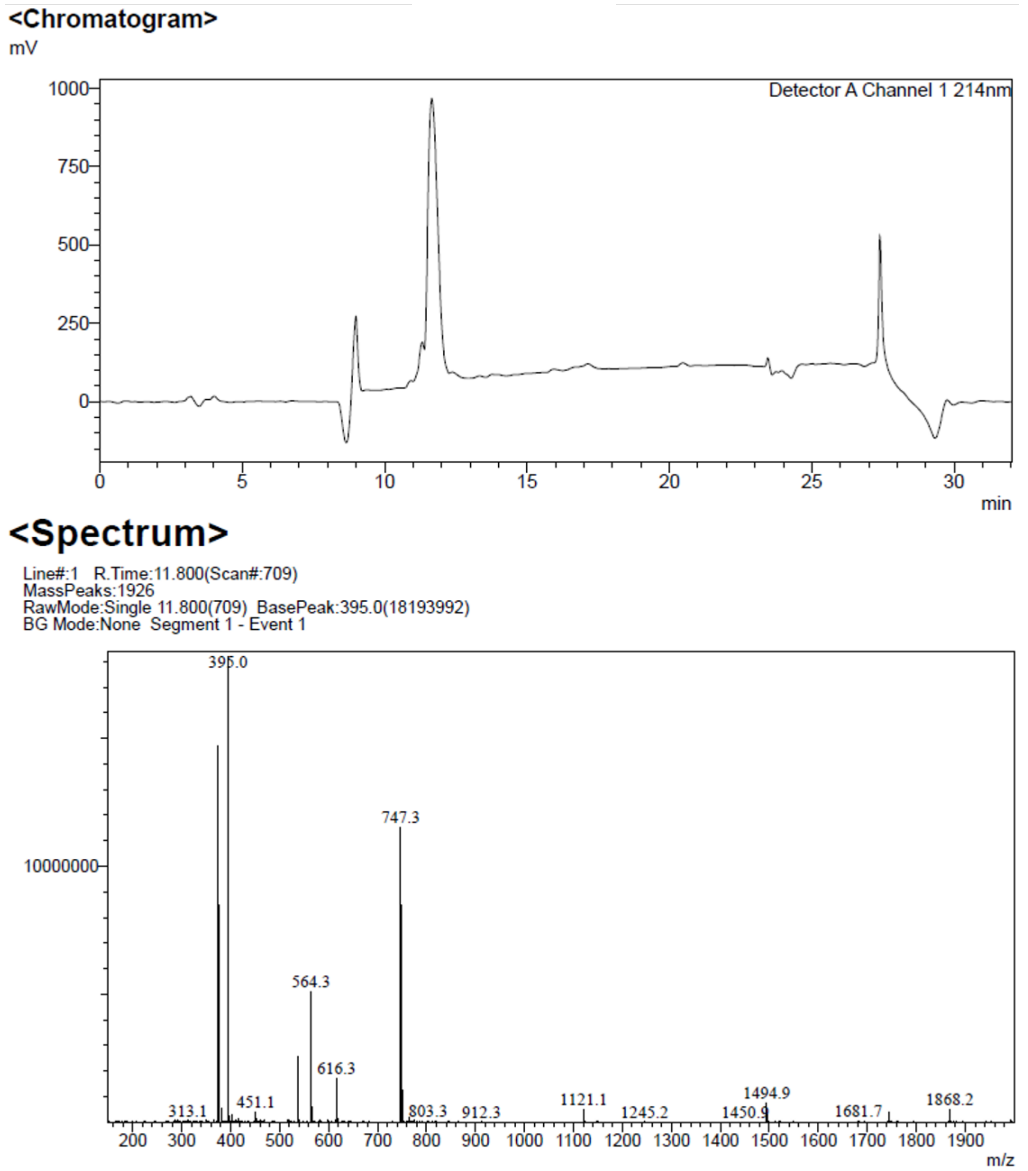

Supporting Figure S5. LC and MS analysis of **3^{5d}**, performed using a 30 min solvent gradient from 0% to 95% (5-25 min) ACN in water (+ 0.1% FA) with a flow rate of 1 mL/min.

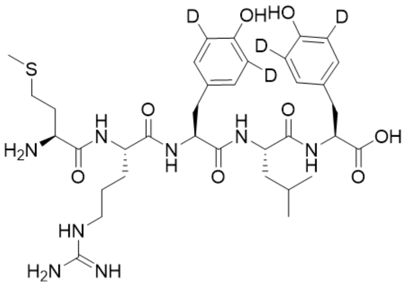

Chemical Formula: $C_{35}H_{48}D_4N_8O_8S$
Exact Mass: 748.38799
Molecular Weight: 748.93341

$3^{3/5d}$

<Chromatogram>

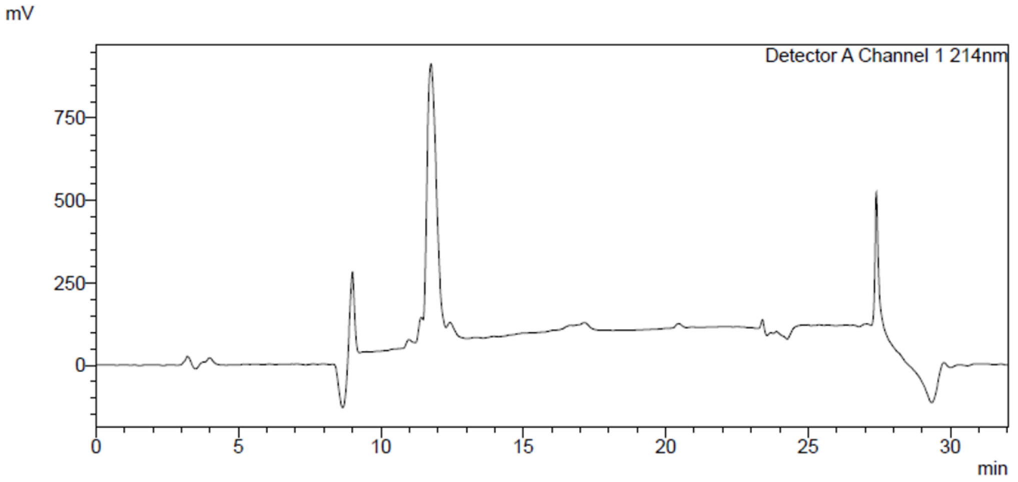

<Spectrum>

Line#:1 R.Time:11.900(Scan#:715)
MassPeaks:1966
RawMode:Single 11.900(715) BasePeak:396.0(20042798)
BG Mode:None Segment 1 - Event 1

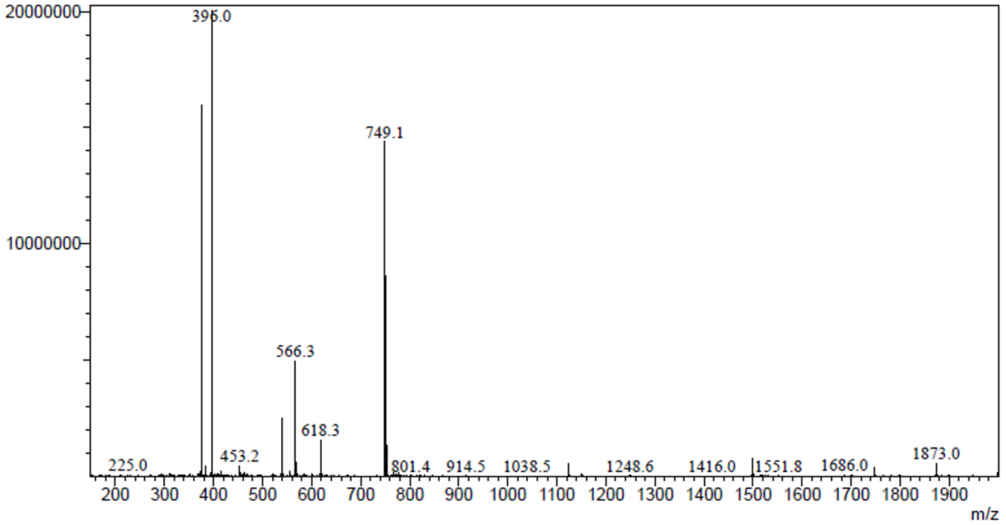

Supporting Figure S6. LC and MS analysis of **$3^{3/5d}$**, performed using a 30 min solvent gradient from 0% to 95% (5-25 min) ACN in water (+ 0.1% FA) with a flow rate of 1 mL/min.

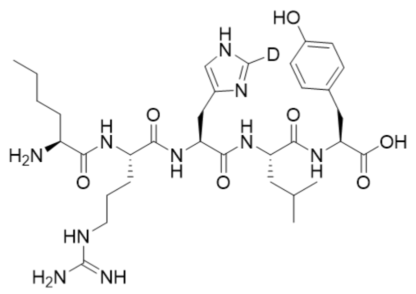

Chemical Formula: $C_{33}H_{51}DN_{10}O_7$
Exact Mass: 701.40832

Nle-$\mathbf{2}^{3d}$

<Chromatogram>

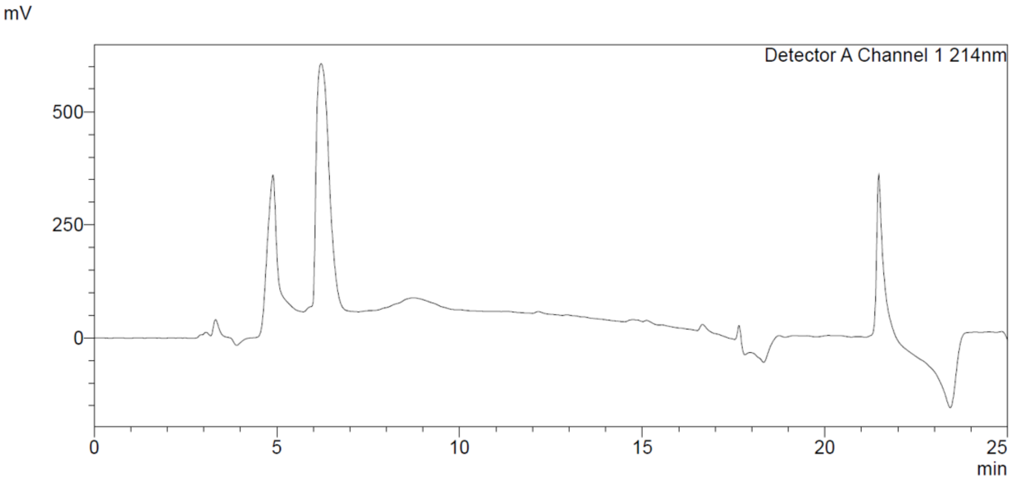

<Spectrum>

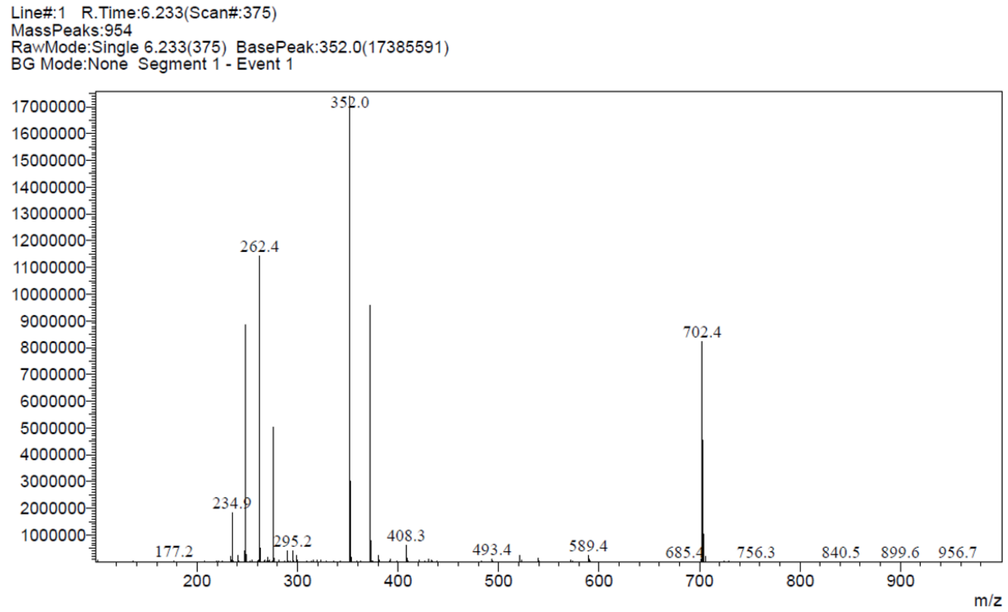

Supporting Figure S7. LC and MS analysis of **Nle-2**$^{\mathbf{3d}}$ **(Nle-R-His(C2-d)-LY)**, performed using a 30 min solvent gradient from 0% to 95% (5-25 min) ACN in water (+ 0.1% FA) with a flow rate of 1 mL/min.

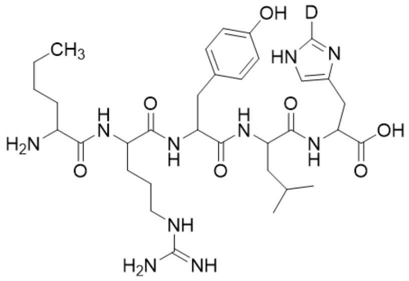

Chemical Formula: $C_{33}H_{51}DN_{10}O_7$
Exact Mass: 701.40832

Nle-1[5d]

<Chromatogram>

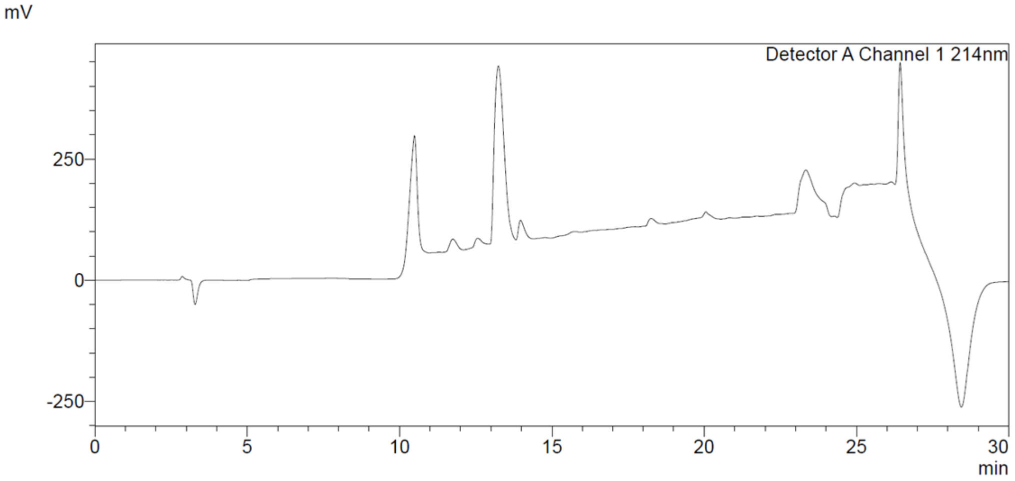

<Spectrum>

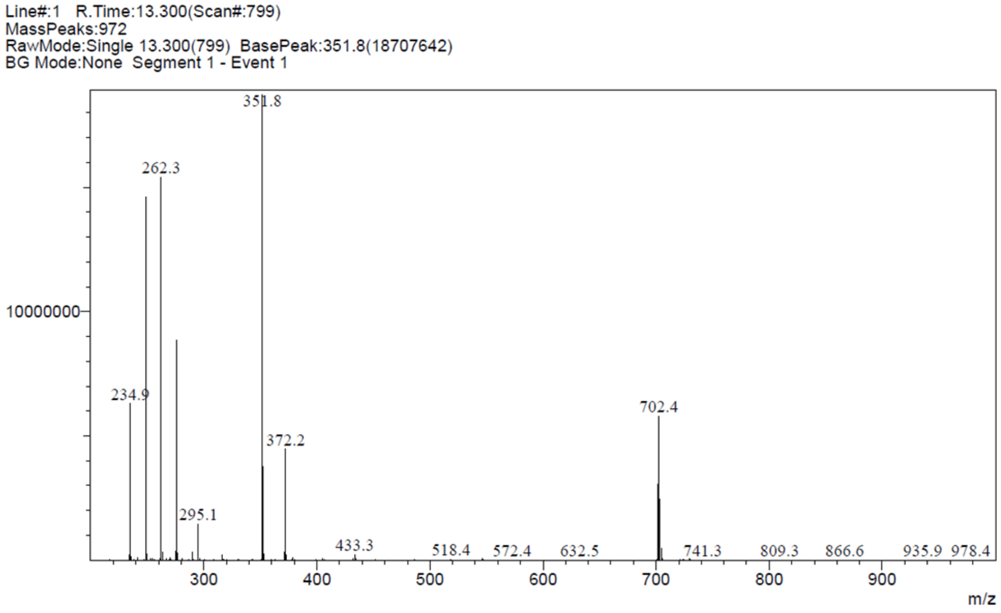

Supporting Figure S8. LC and MS analysis of **Nle-1[5d] (Nle-RYL-His(C2-d))**, performed using a 30 min solvent gradient from 0% to 95% (5-25 min) ACN in water (+ 0.1% FA) with a flow rate of 1 mL/min.

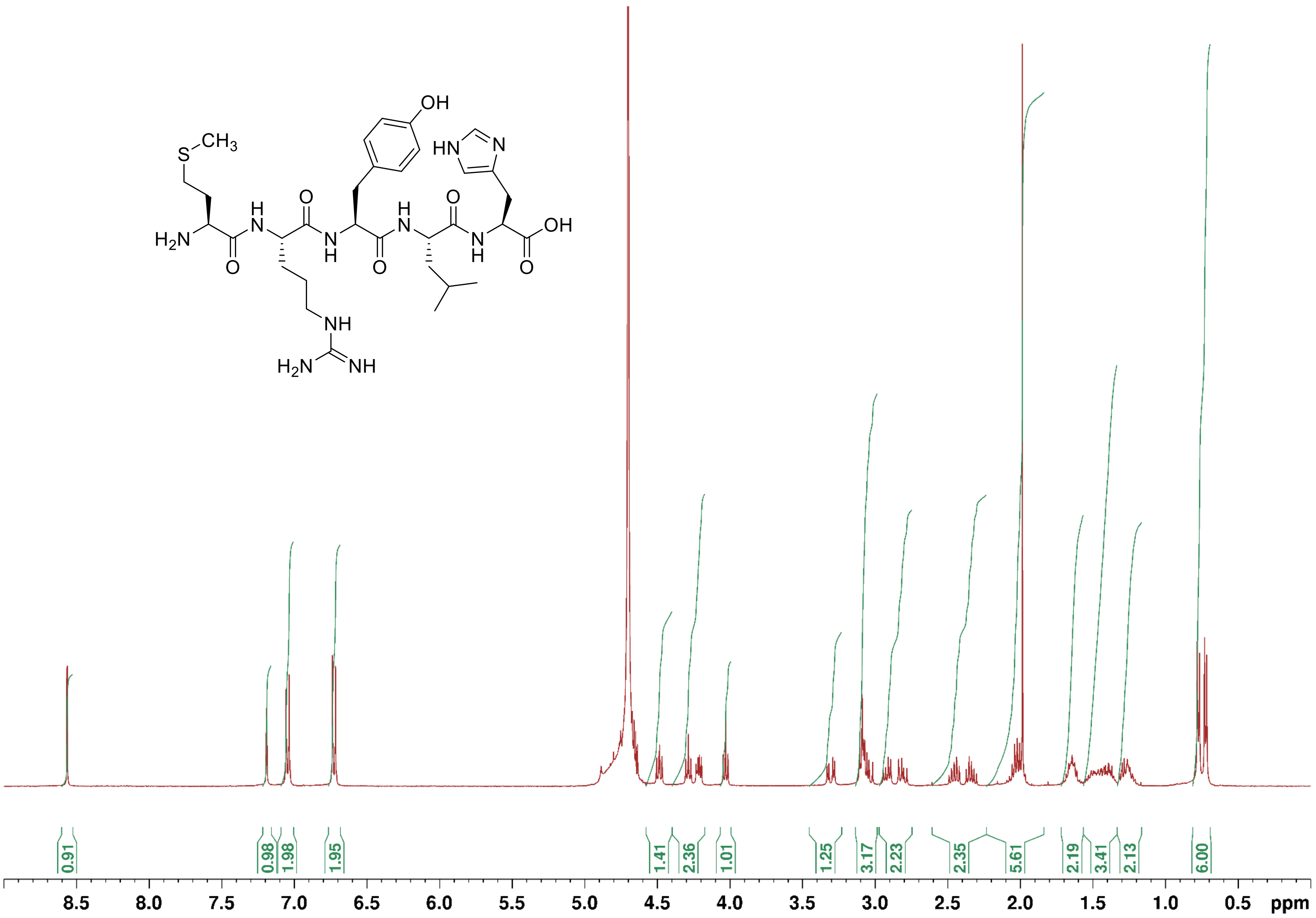

Supporting Figure S9. ^{1}H NMR spectra for 1 (400 MHz, D_2O).

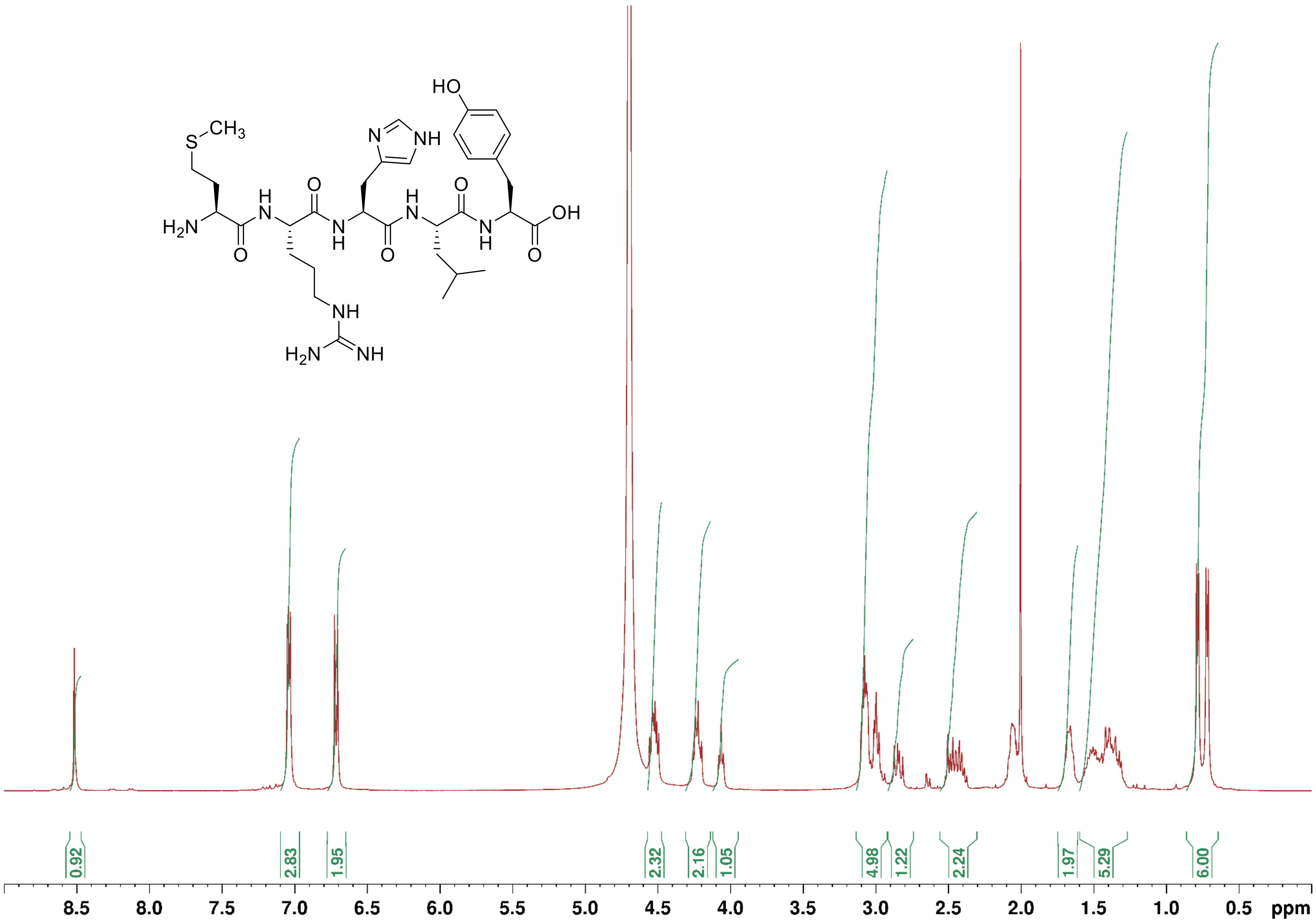

Supporting Figure S10. ^{1}H NMR spectra for 2 (400 MHz, D_2O).

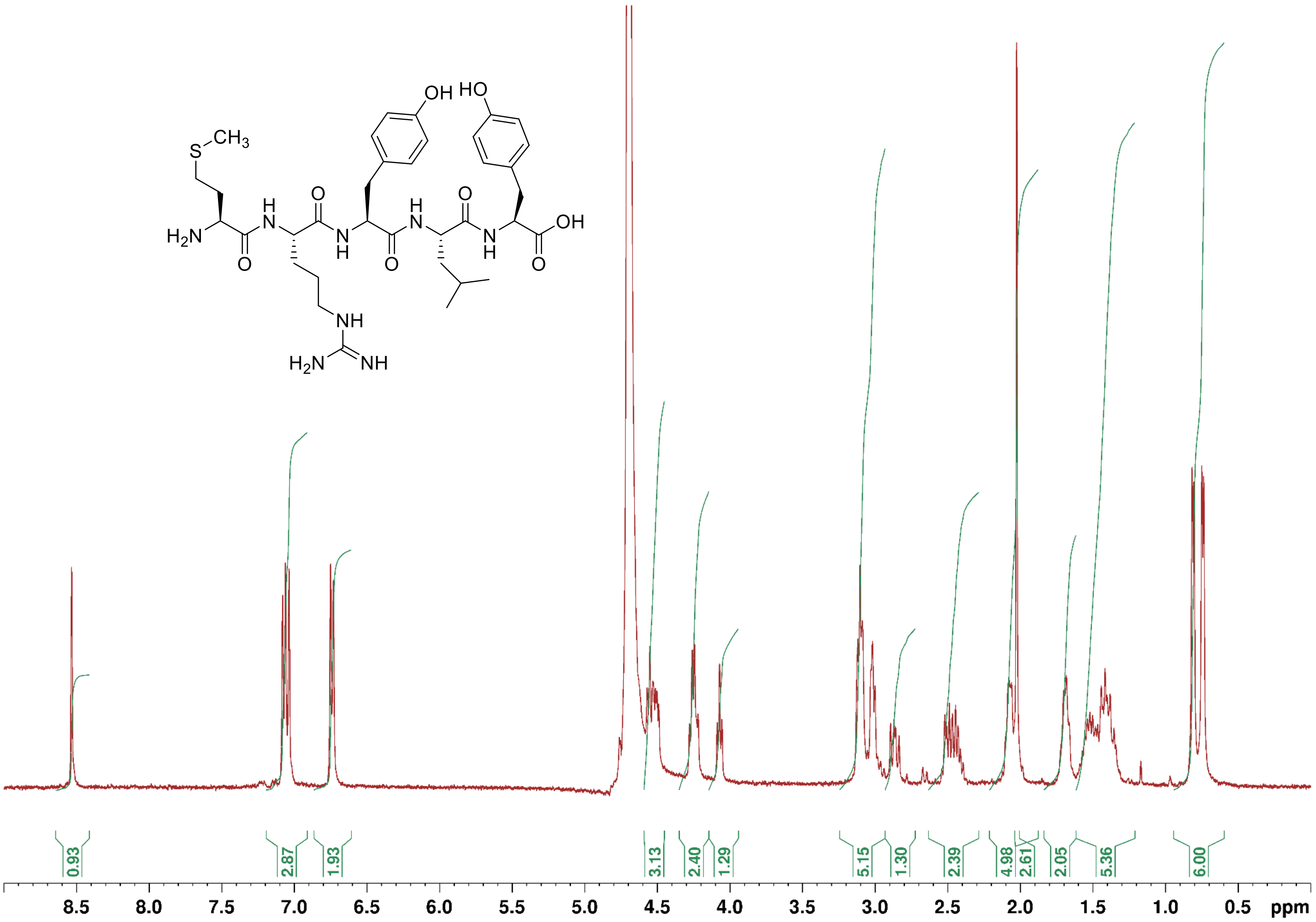

Supporting Figure S11. ^{1}H NMR spectra for 3 (400 MHz, D_2O).

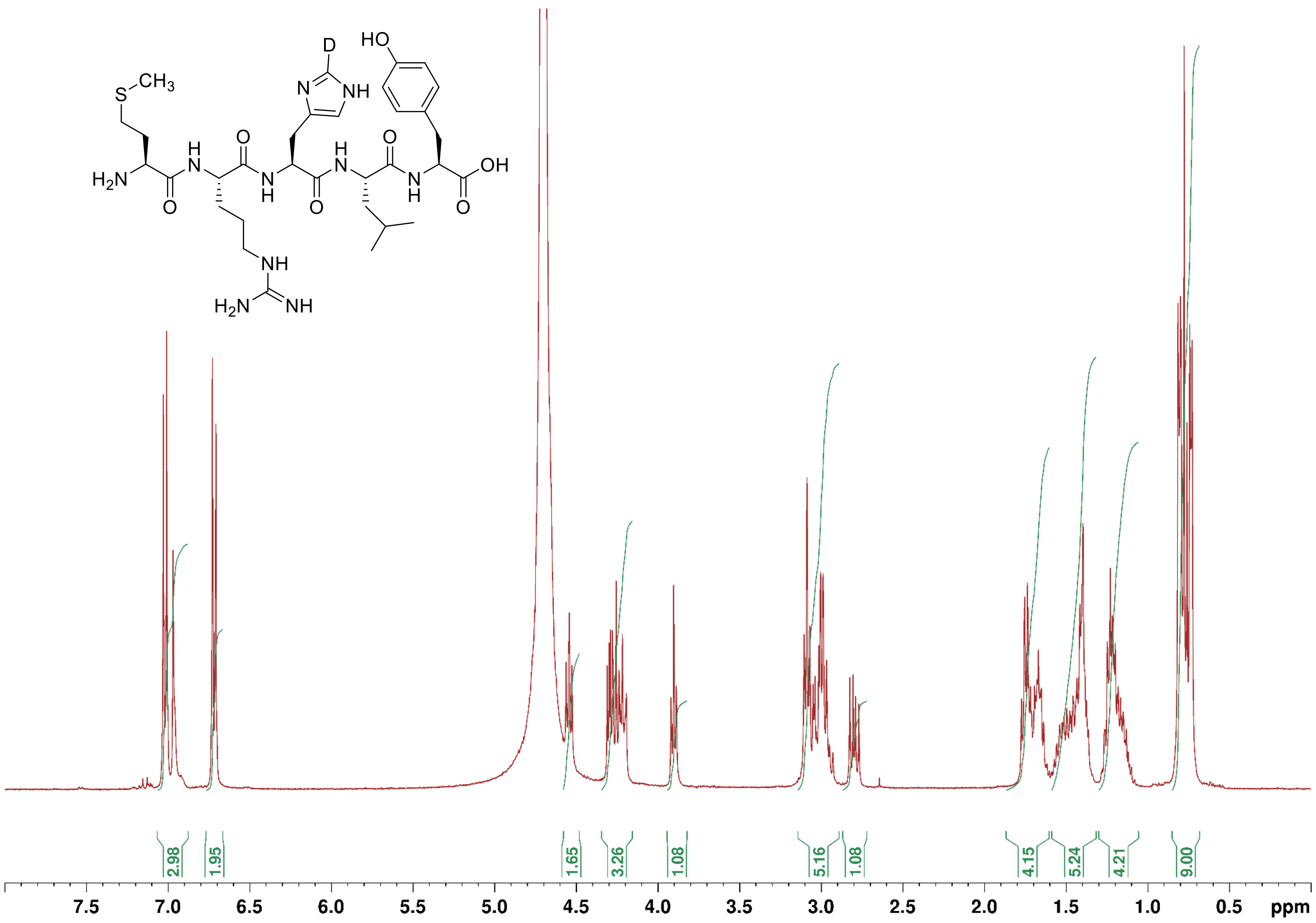

Supporting Figure S12. ^{1}H NMR spectra for Nle-2^{3d} (Nle-R-His(C2-d)-LY, 400 MHz, D_2O).

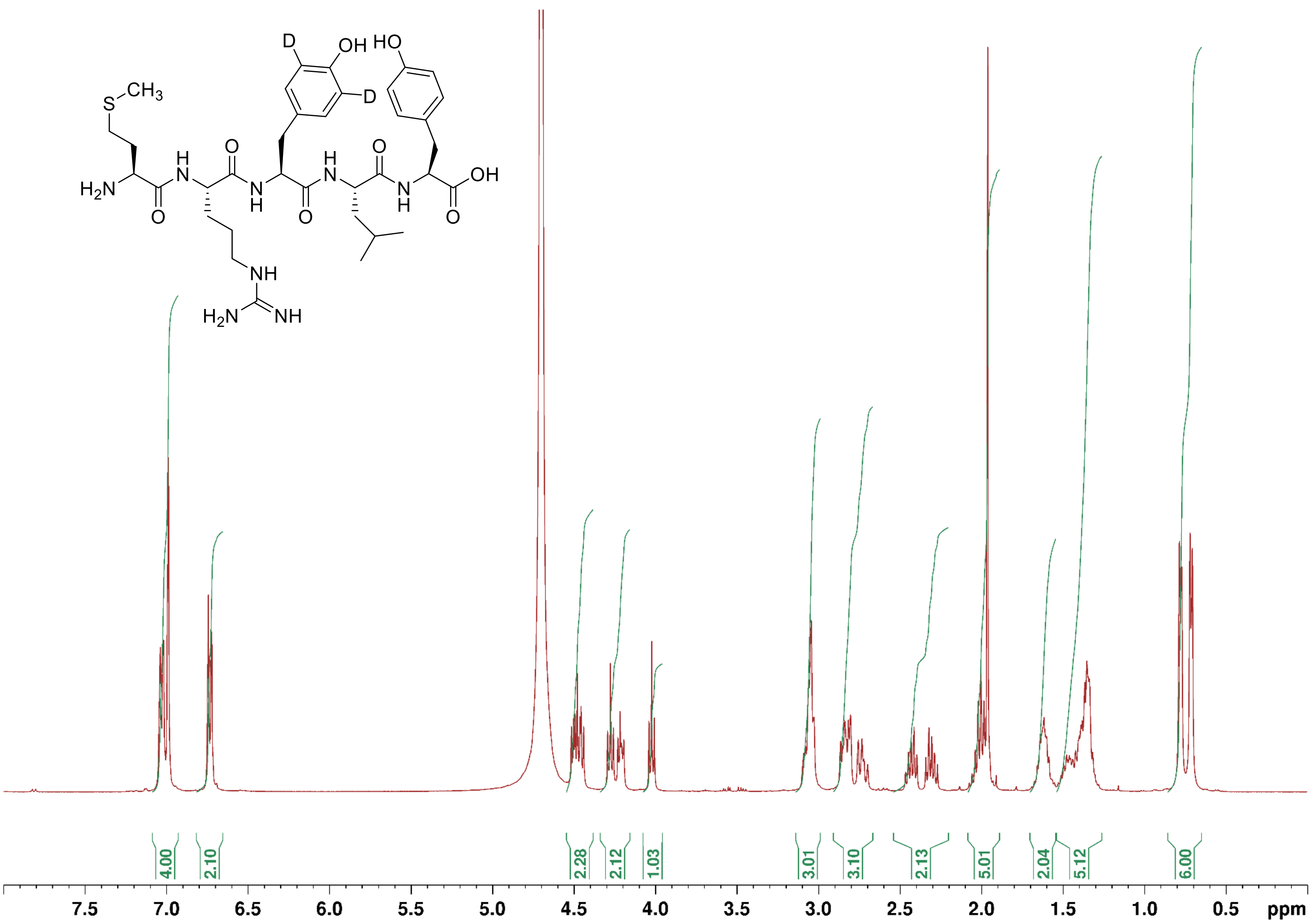

Supporting Figure S13. **^{1}H NMR spectra for 3[3d] (400 MHz, D_2O).**

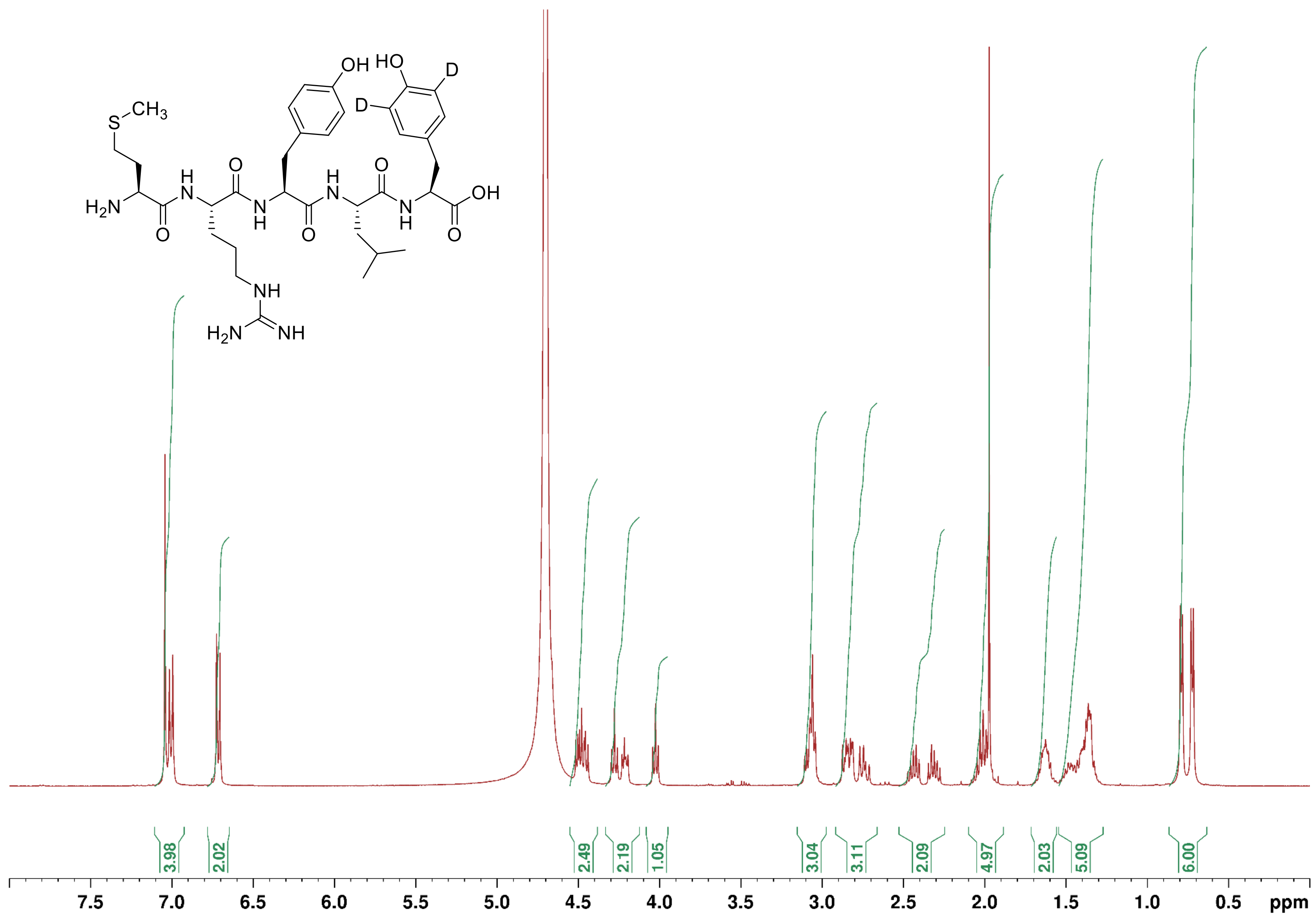

Supporting Figure S14. ^{1}H NMR spectra for 3^{5d} (400 MHz, D_2O).

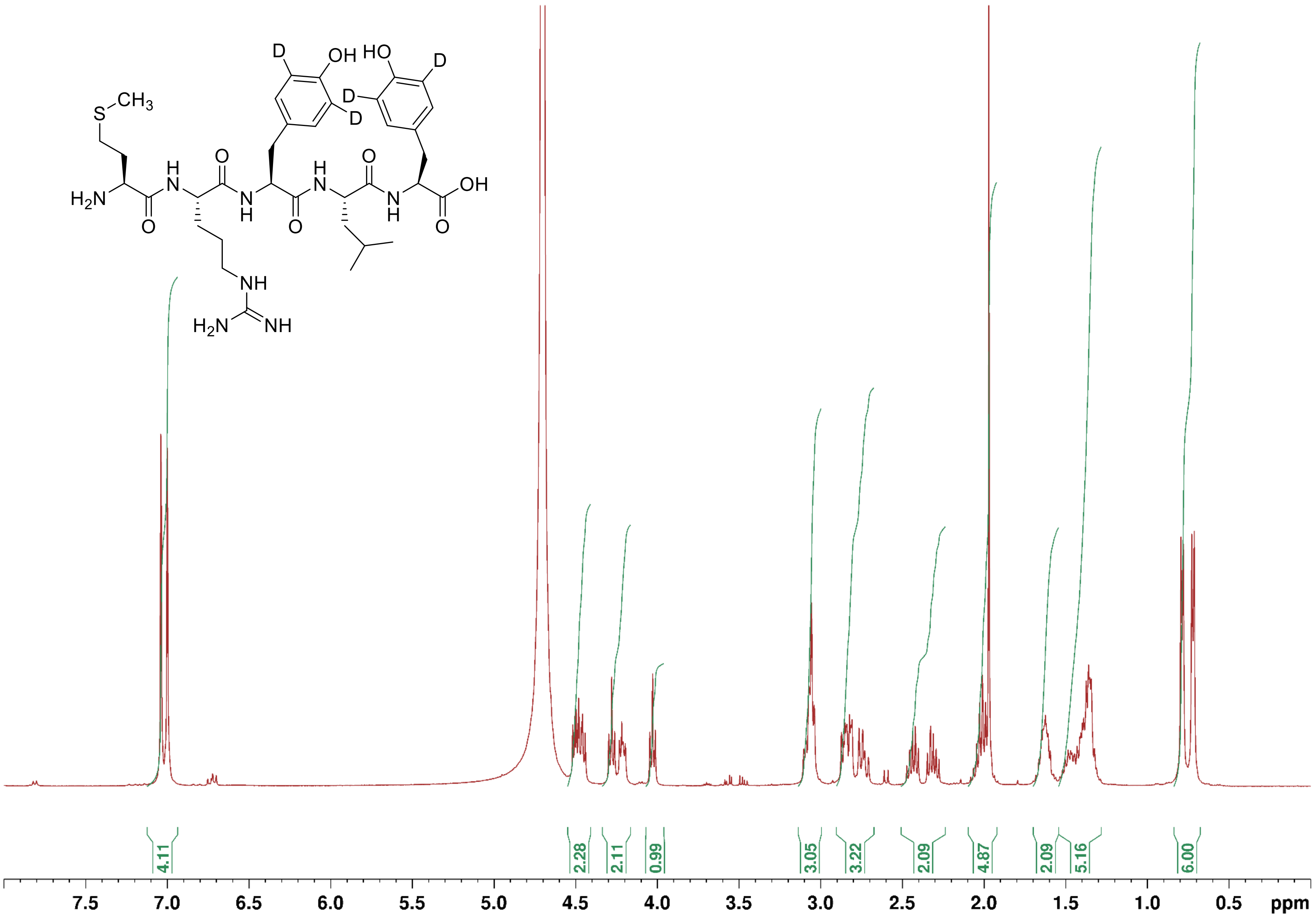

Supporting Figure S15. ^{1}H NMR spectra for $3^{3/5d}$ (400 MHz, D_2O).

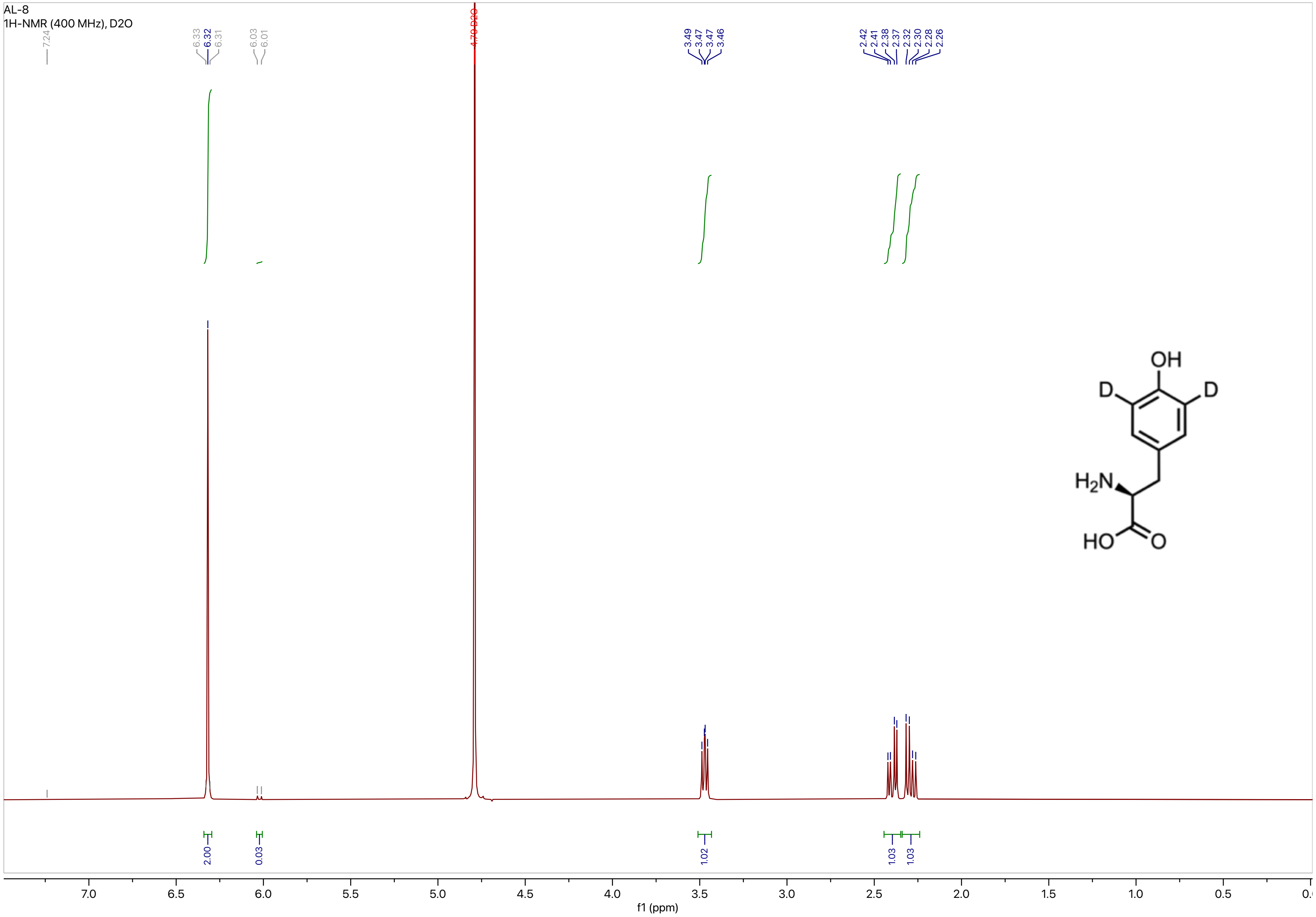

Supporting Figure S16. **^{1}H-NMR spectrum of L-tyrosine-(*phenyl*-3,5-d$_2$) (400 MHz, D_2O).**

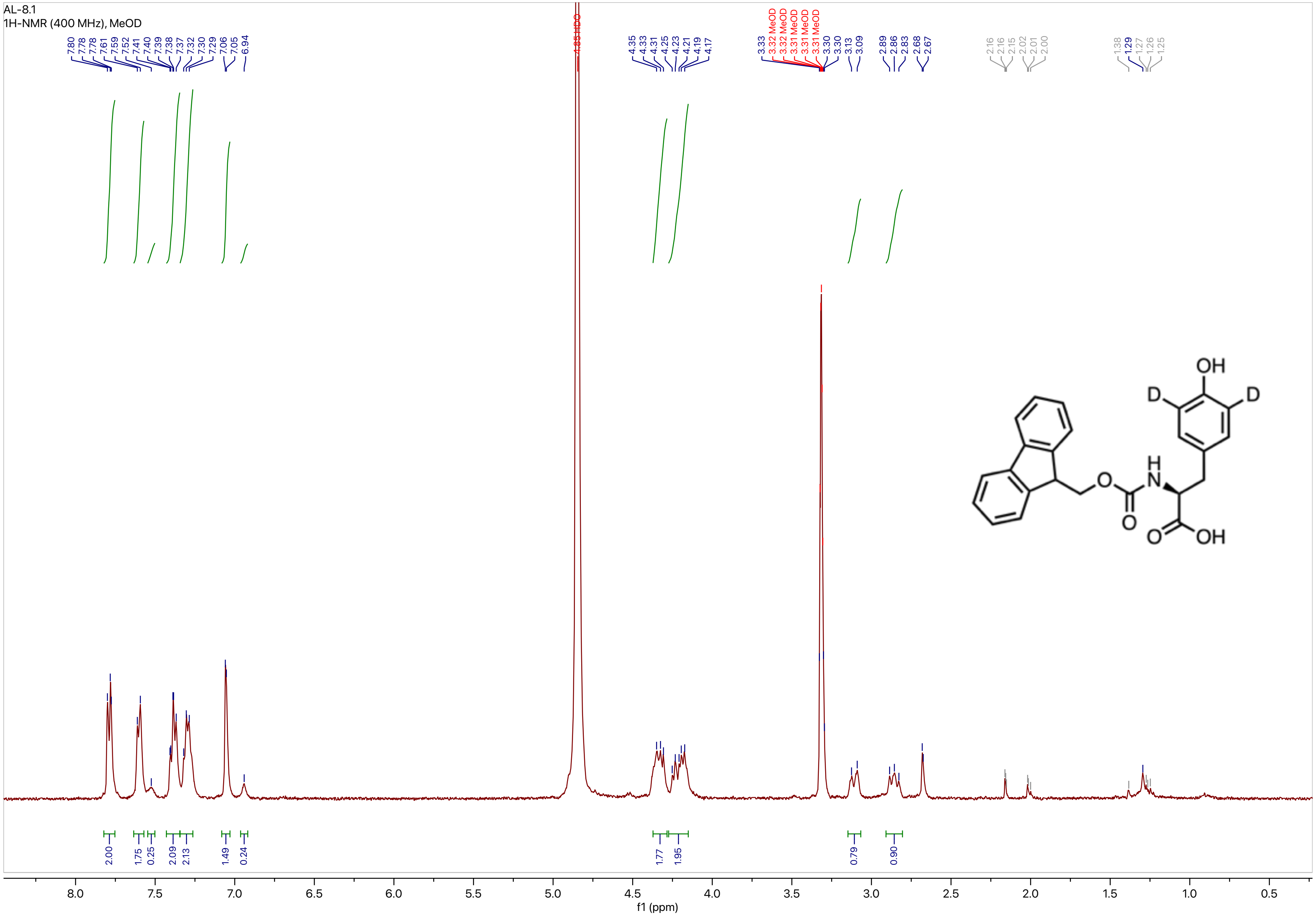

Supporting Figure S17. **^{1}H-NMR of Fmoc-L-tyrosine-(*phenyl*-3,5-d$_2$) (400 MHz, CD$_3$OD).**

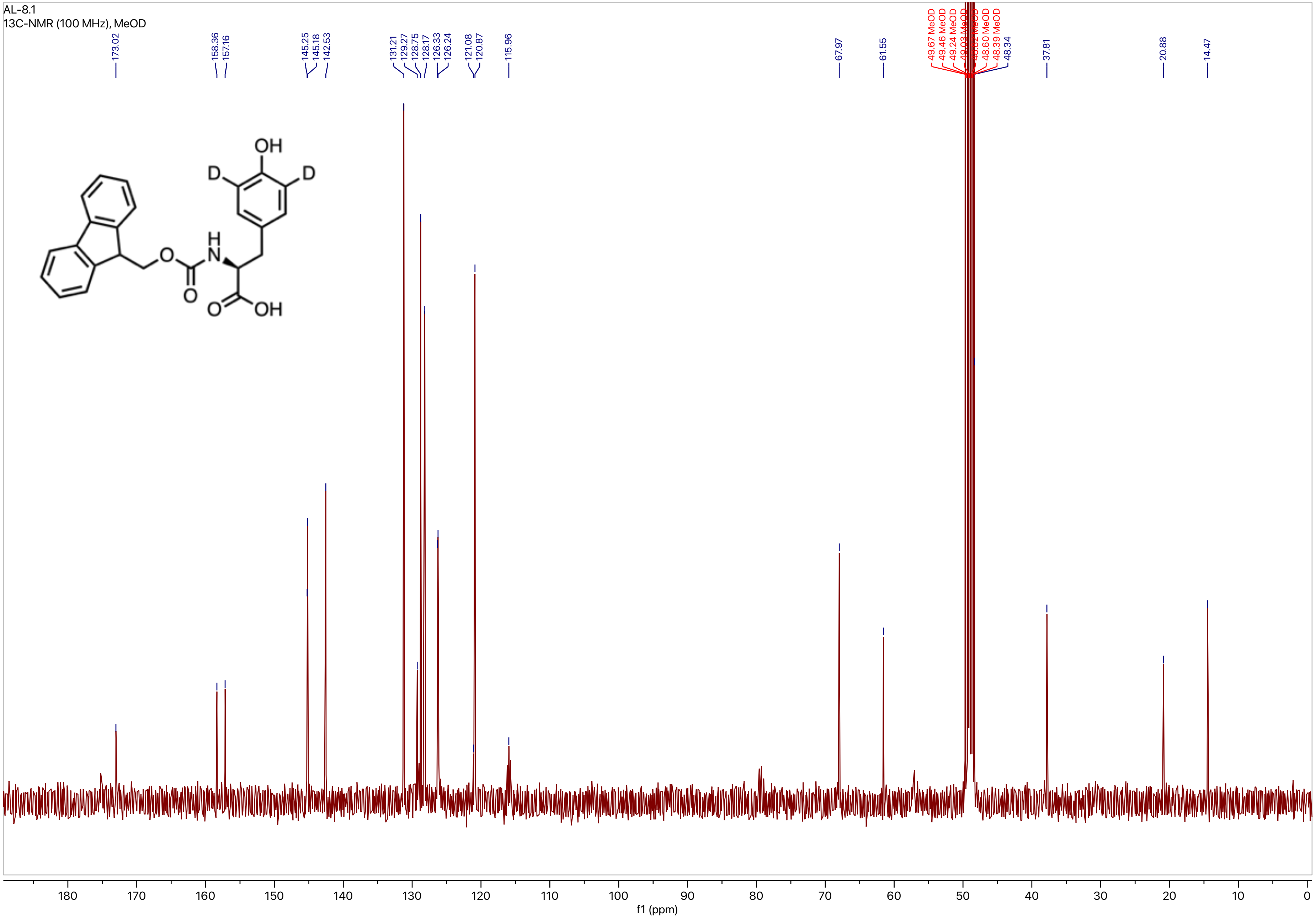

Supporting Figure S18. **^{13}C-NMR spectra of Fmoc-L-tyrosine-(*phenyl*-3,5-d$_2$) (101 MHz, CD$_3$OD).**

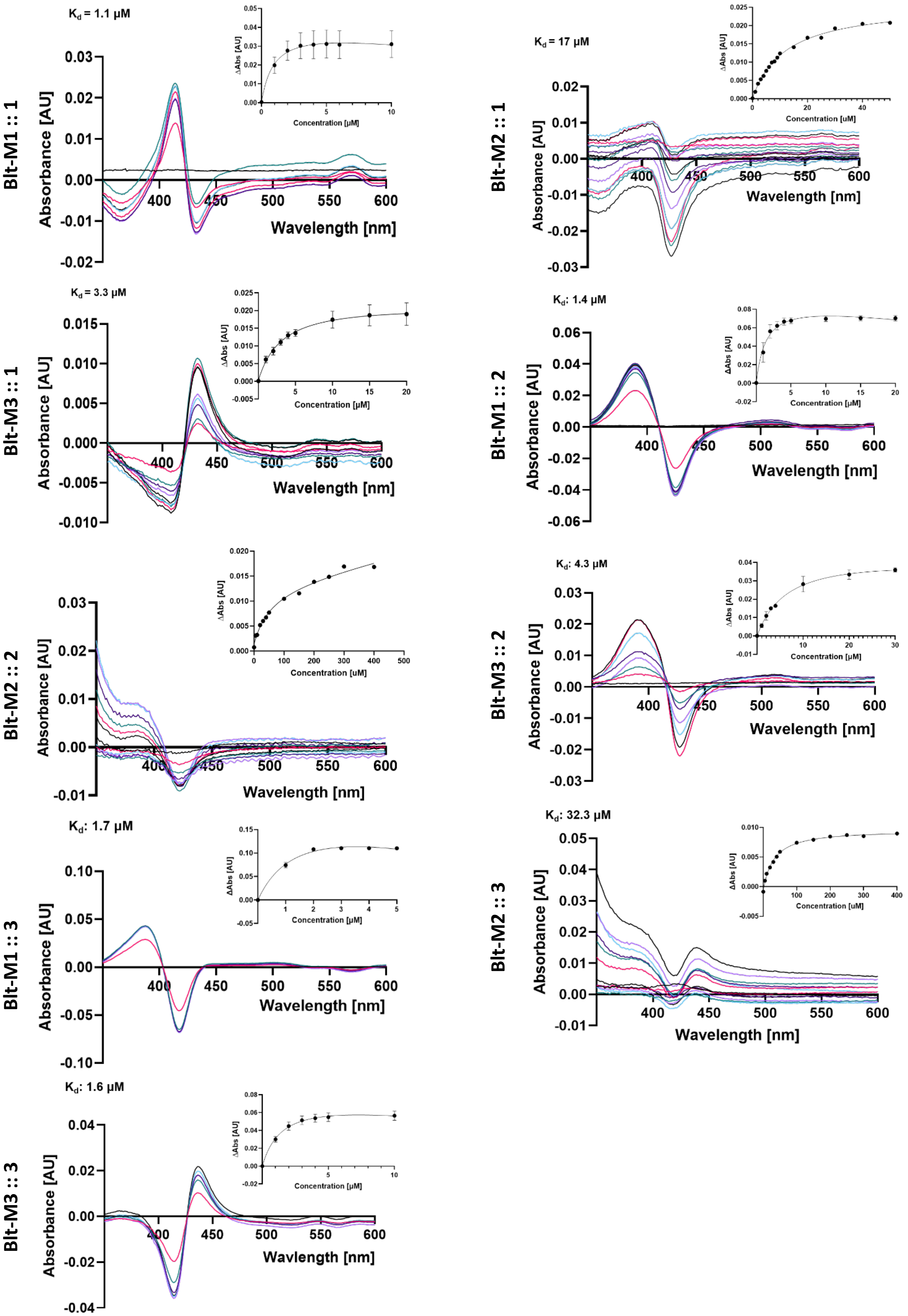

Supporting Figure S19. Assessment of substrate binding to *Blt-M1, Blt-M2* and *Blt-M3* using peptide substrates **1-3** assed by UV/Vis spectroscopy. K_d values calculated as described in the methods section.

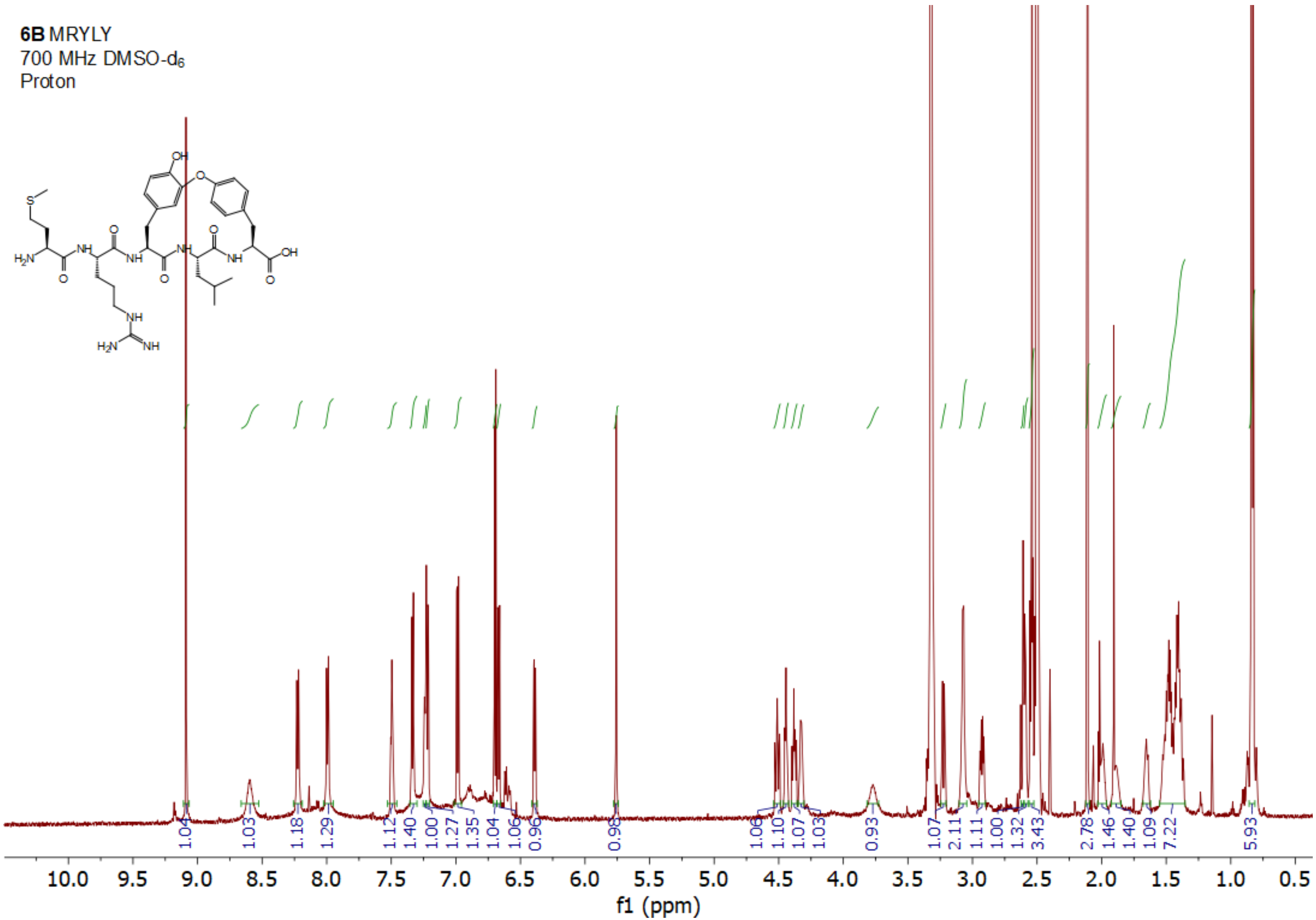

Supporting Figure S20. ^{1}H-NMR spectrum of 6B.

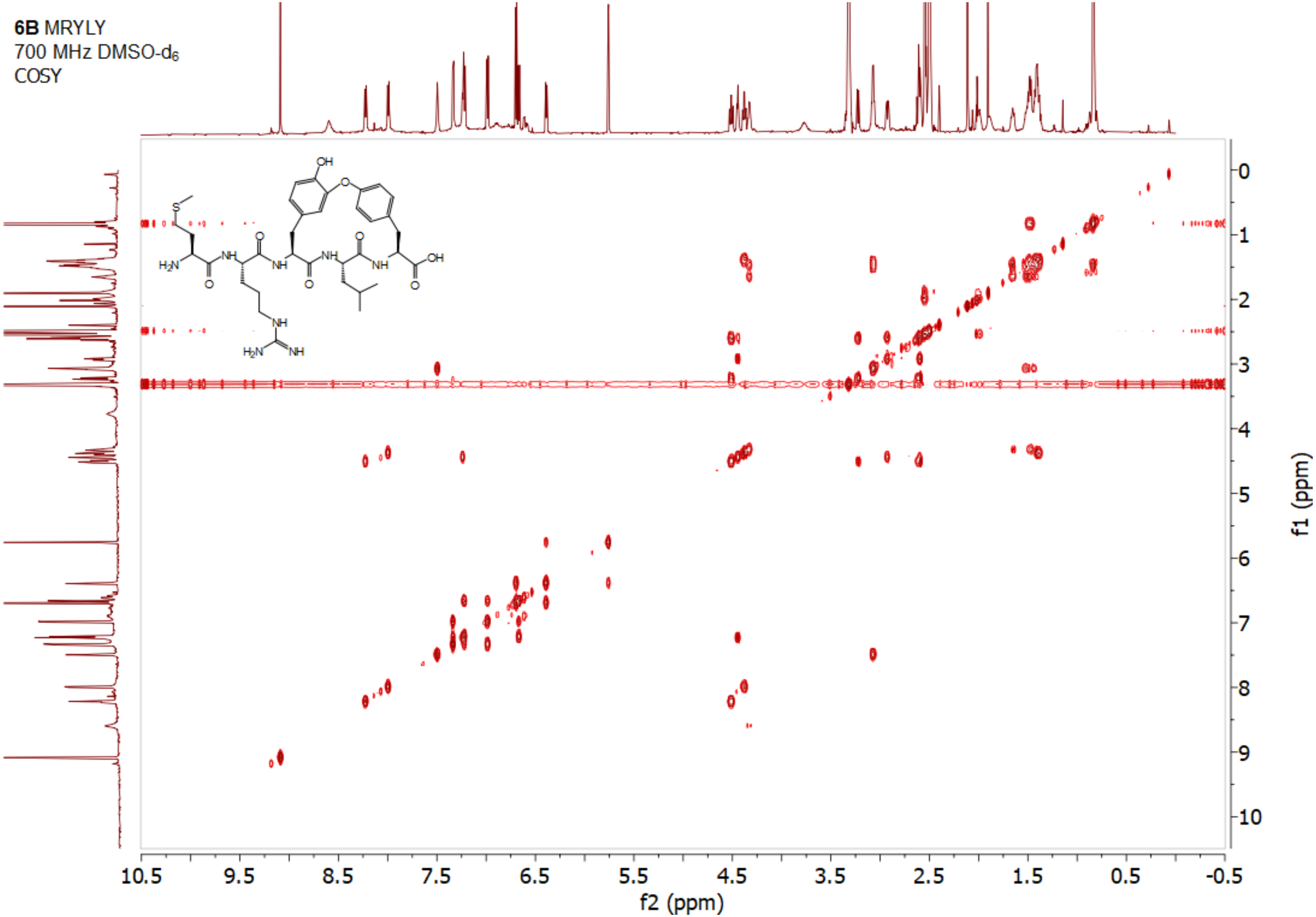

Supporting Figure S21. COSY spectrum of 6B.

6B MRYLY
700 MHz DMSO-d_6
TOCSY

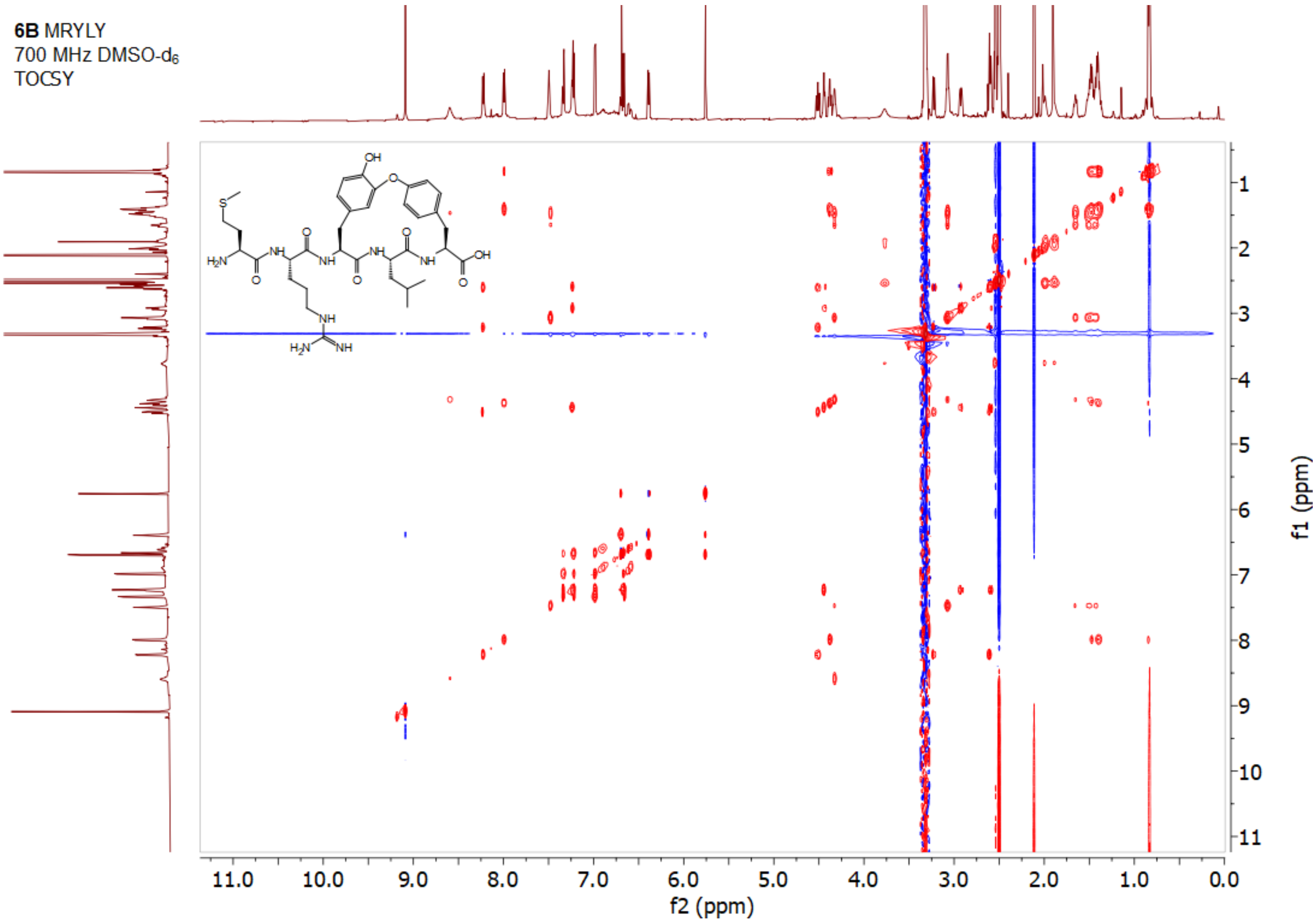

Supporting Figure S22. TOCSY spectrum of 6B.

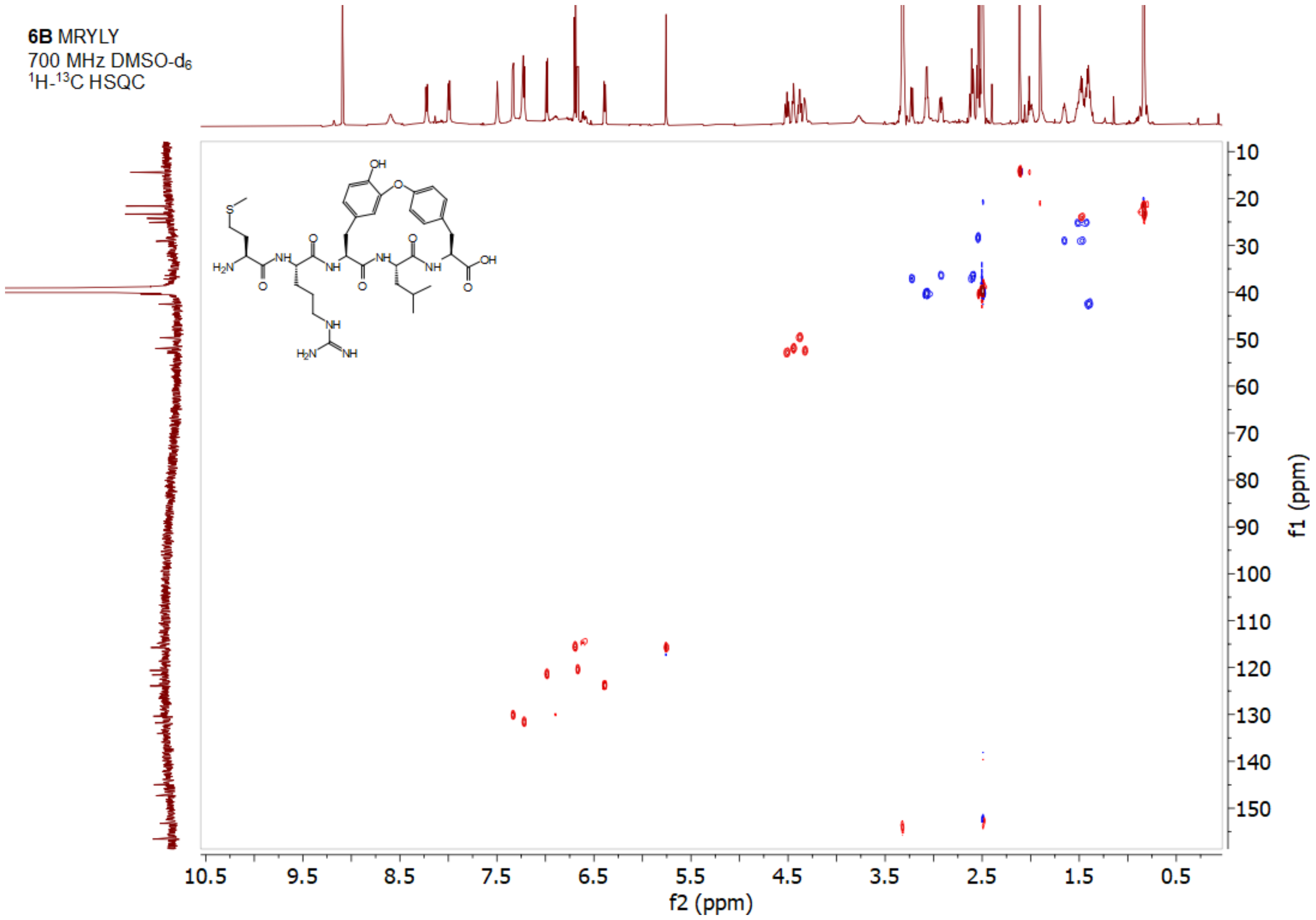

Supporting Figure S23. ^{1}H-^{13}C HSQC spectrum of 6B.

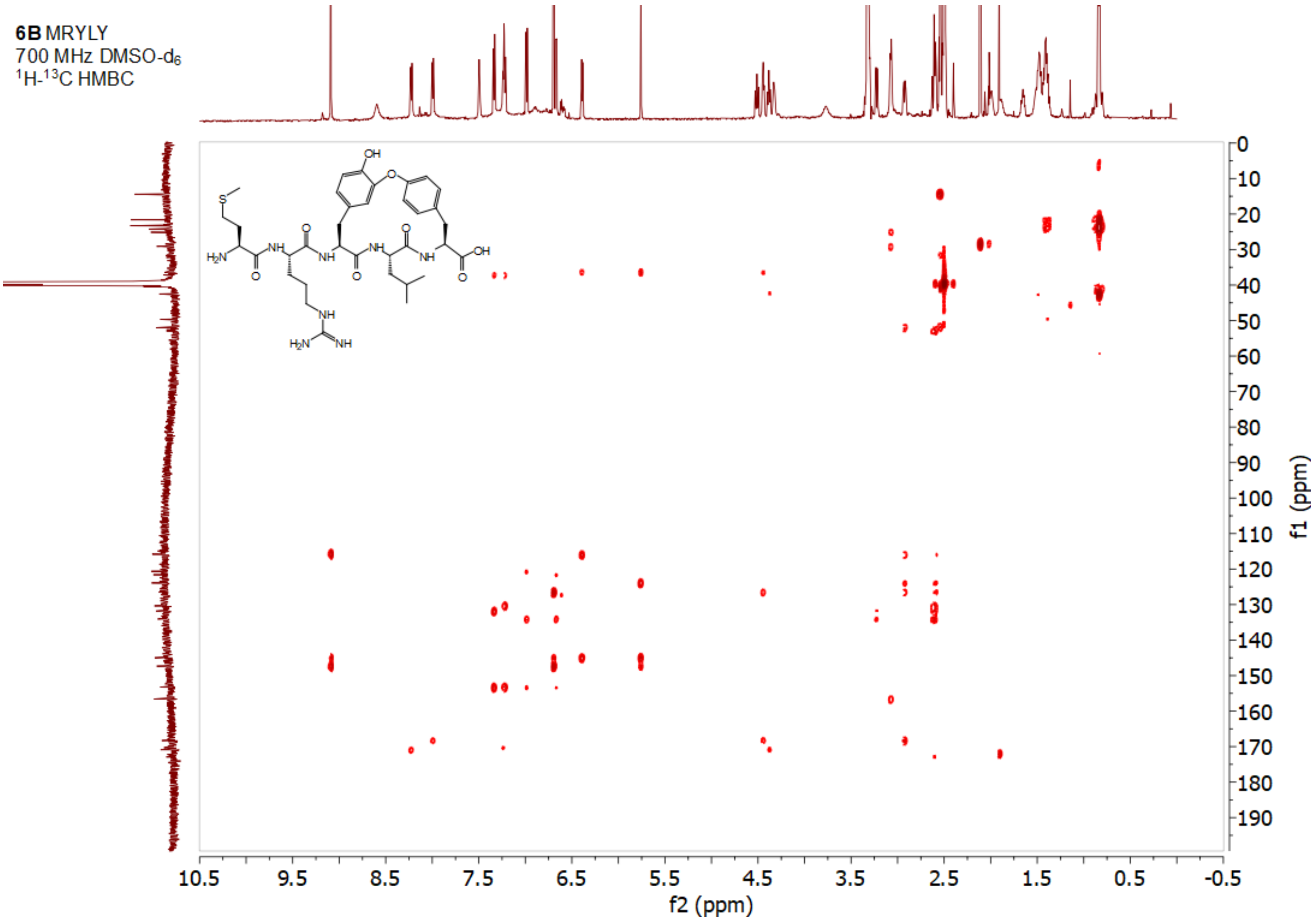

Supporting Figure S24. ^{1}H-^{13}C HMBC spectrum of 6B.

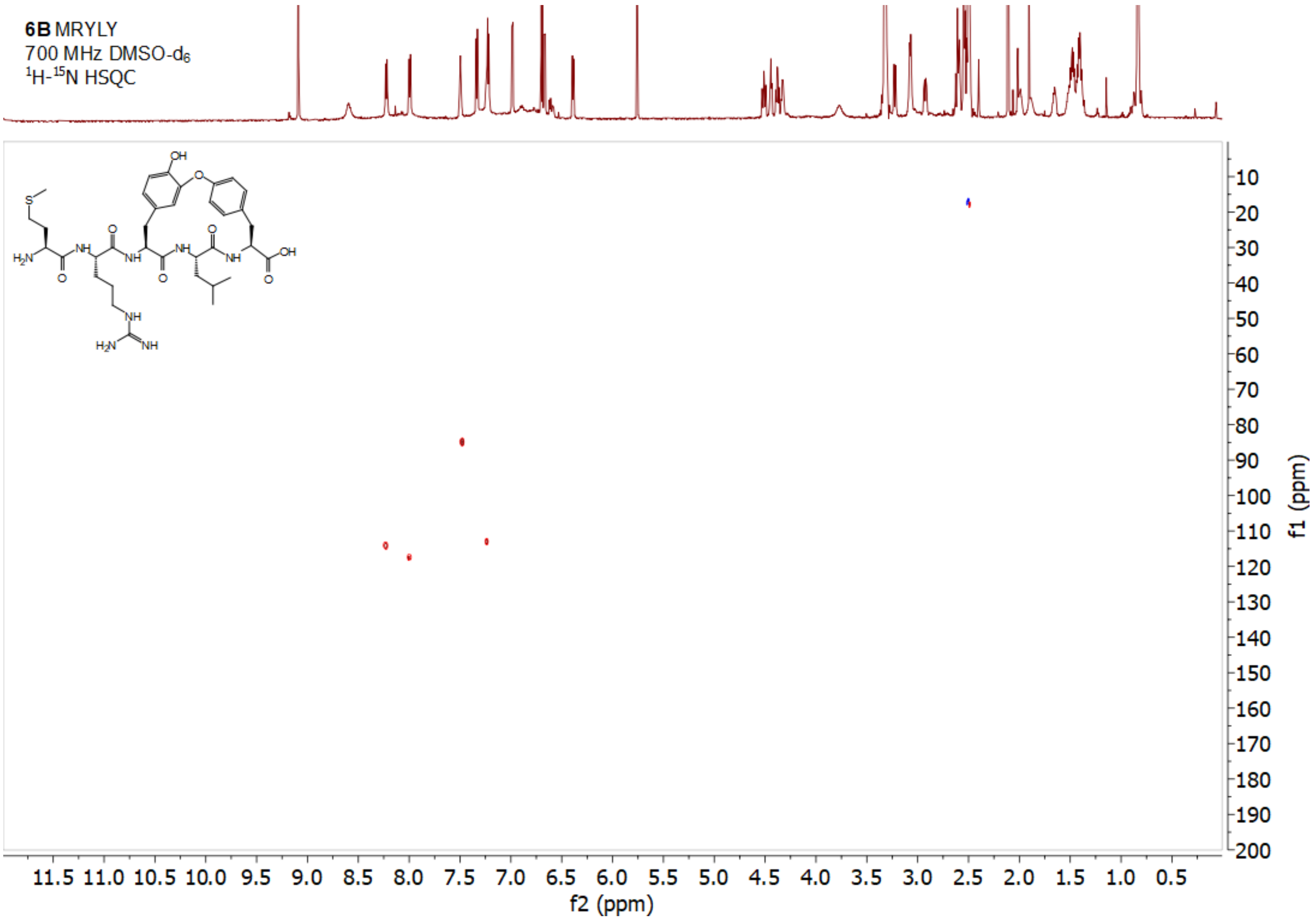

Supporting Figure S25. ^{1}H-^{15}N HSQC spectrum of 6B.

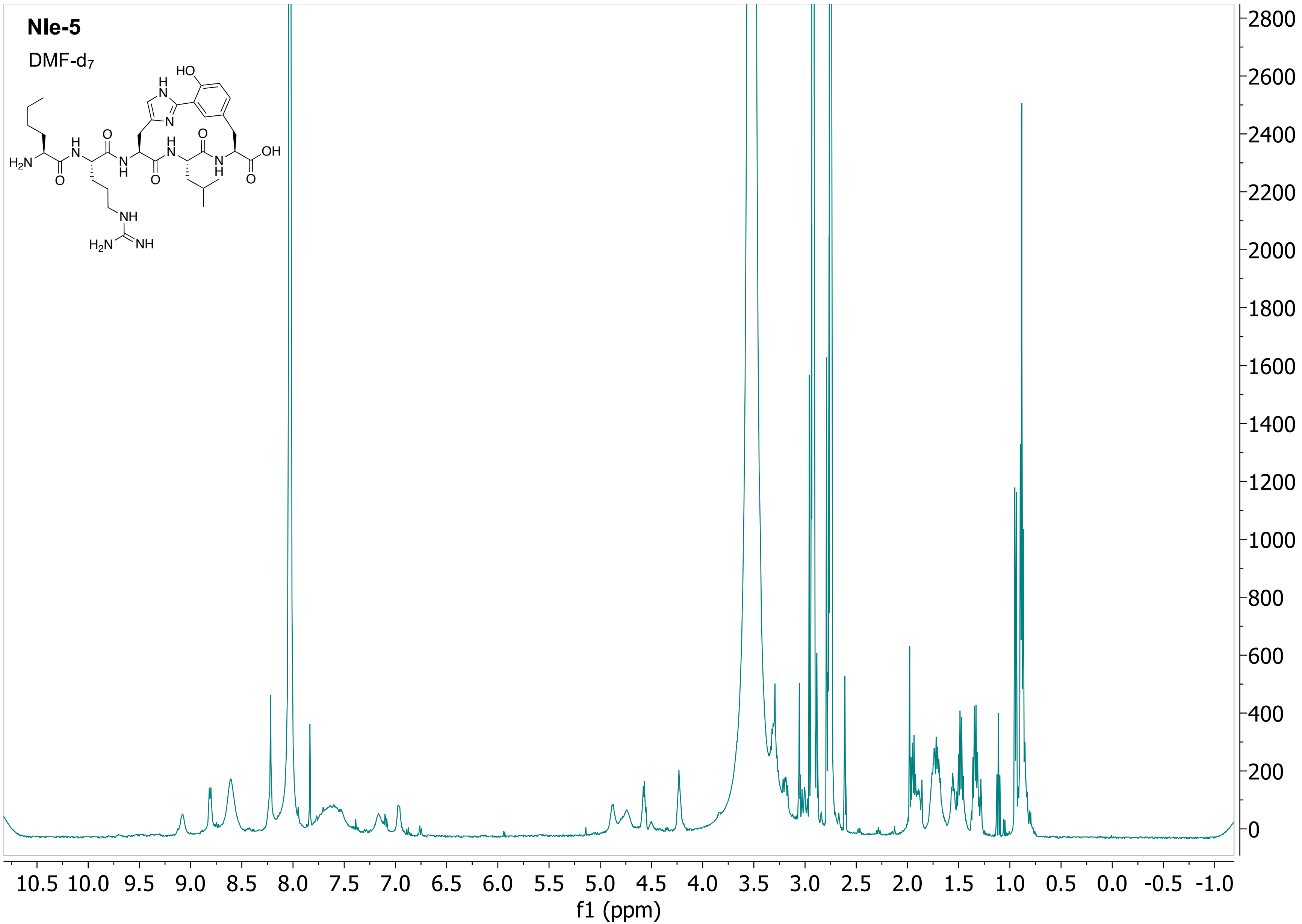

Supporting Figure S26. ^{1}H-NMR spectrum of **5**

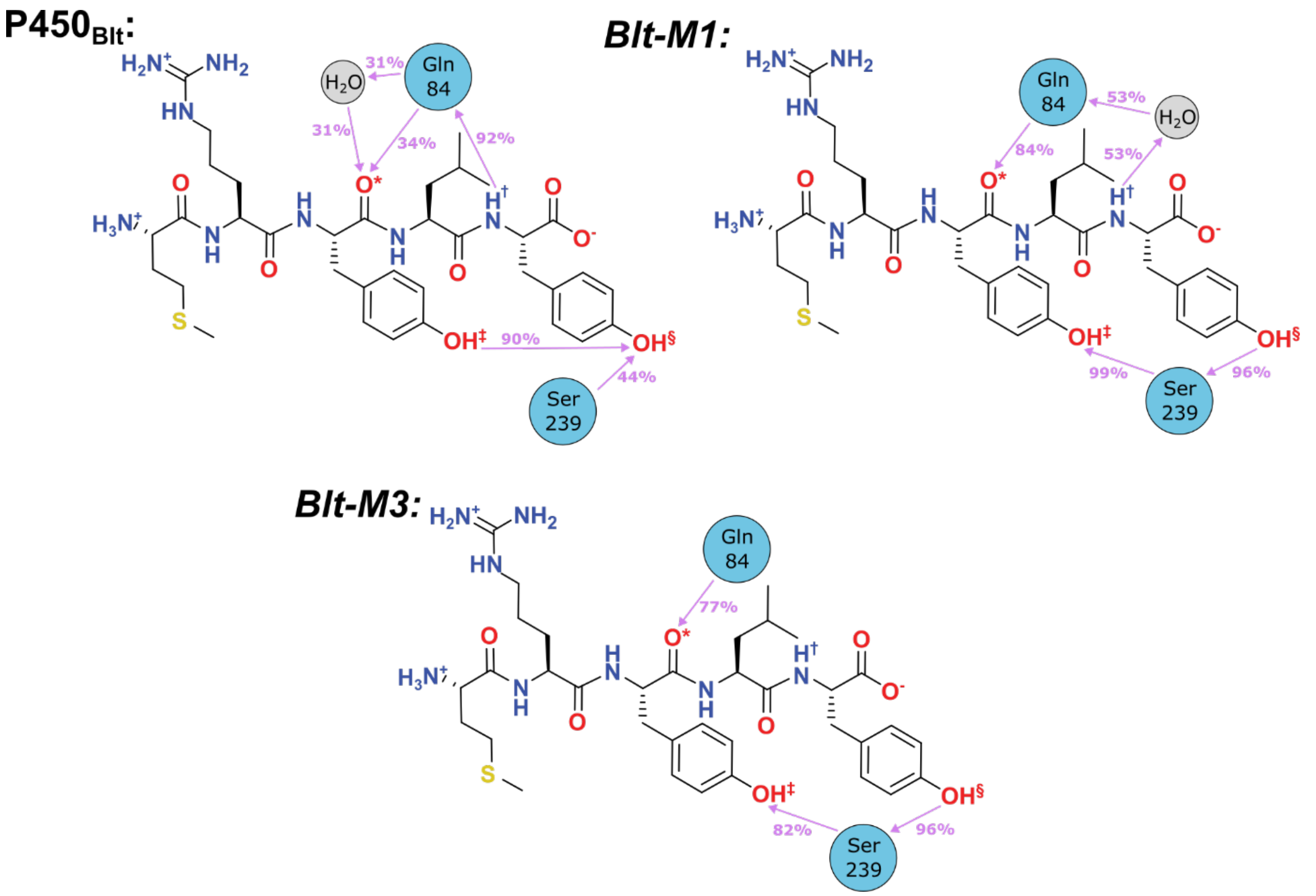

	P450$_{Blt}$			*Blt-M1*			*Blt-M3*		
	SIM 1	SIM 2	SIM 3	SIM 1	SIM 2	SIM 3	SIM 1	SIM 2	SIM 3
					**Gln84**				
MRYLY Leu$_4$/Tyr$_5$ amide (*)	0 (67 W)	92	94	54	0 (53 W)	0 (54 W)	64 (31 W)	0	83
MRYLY Tyr$_3$/Leu$_4$ carbonyl (†)	61	34 (31 W)	0 (31 W)	0 (39 Na)	84	73	52	77	52
					**Ser239**				
Tyr$_3$-OH (‡)	79	0	0	99	99	99	77	82	99
Tyr$_5$-OH (§)	78	44	68	96	96	95	81	96	93
					**MRYLY Tyr$_3$-OH(‡)--Tyr$_5$-OH(§)**				
	0	90	52 + 43*	0	0	0	0	0	0

Supporting Figure S27: Molecular dynamics simulation indicating the interactions of docked MRYLY peptide with P450$_{Blt}$ and mutants *Blt-M1* and *Blt-M3*. Schematic representation of the interactions between P450$_{Blt}$ residues Gln84 and Ser239 to docked MRYLY. Purple arrows indicate hydrogen bond interaction with percentage of frames the interaction is observed in over one 200 ns molecular dynamics simulation. Molecular dynamics simulations were conducted in triplicate (SIM 1-3) and the Gln84, Ser239 and intramolecular hydrogen bonding between the MRYLY tyrosine residues (acceptor and donor interactions) are indicated in the table (% number of frames, W: water mediated, Na: sodium-mediated).

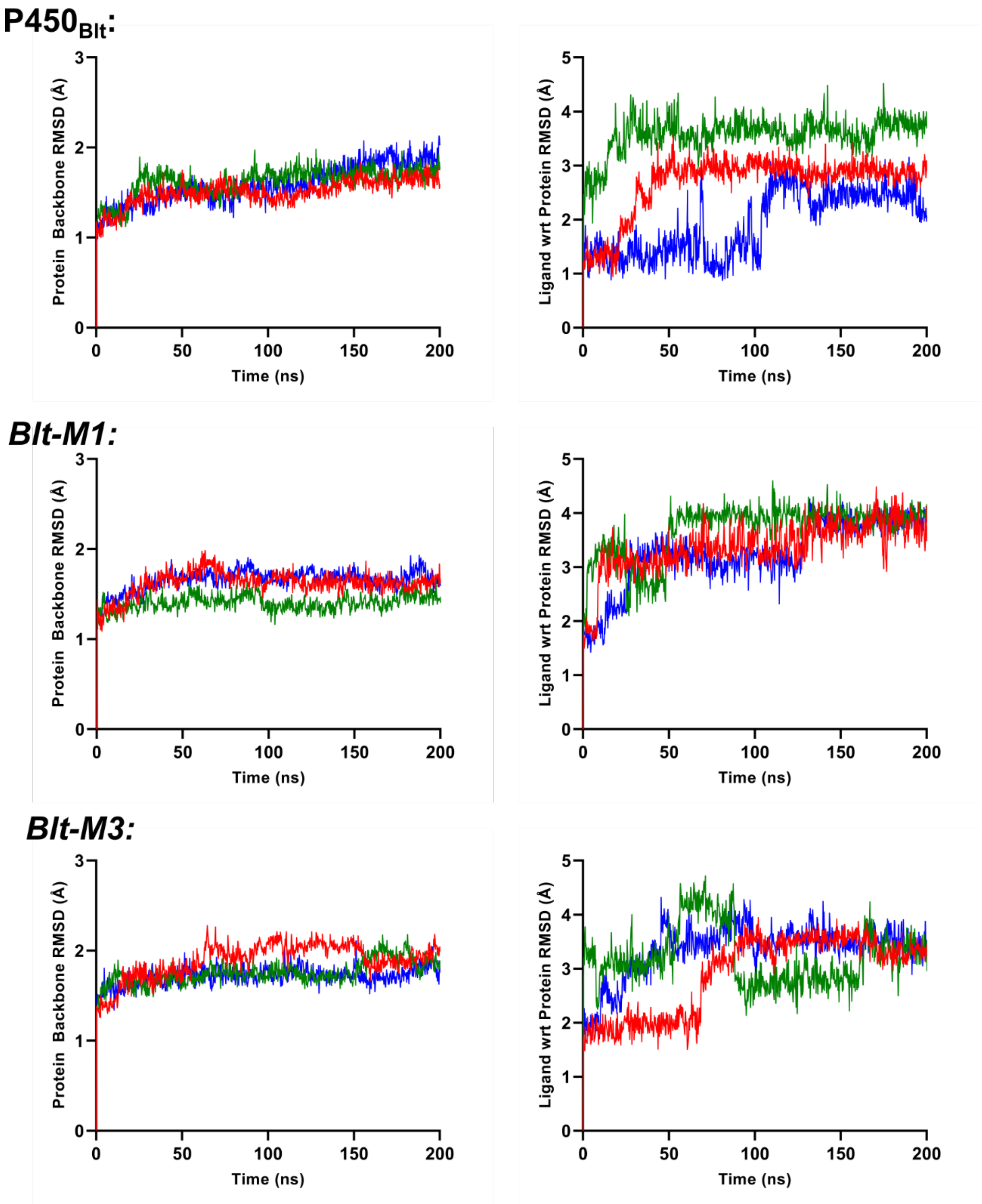

Supporting Figure S28: Molecular dynamics simulations showing the stability of the complex of docked MRYLY peptide with P450$_{Blt}$ and mutants *Blt-M1* and *Blt-M3*. 200 ns molecular dynamics simulations were undertaken in triplicate (Red: SIM 1, Green: SIM 2, Blue: SIM 3) for the docked MRYLY-P450$_{Blt}$ complex. Protein backbone and ligand with respect to protein backbone Root Mean Square Deviation (RMSD) is relative to the initial frame. RMSD of the protein backbone indicate a stable conformation is obtained after 25-50 ns. RMSD of the ligand with respect to protein is stable after 150 – 175 ns.

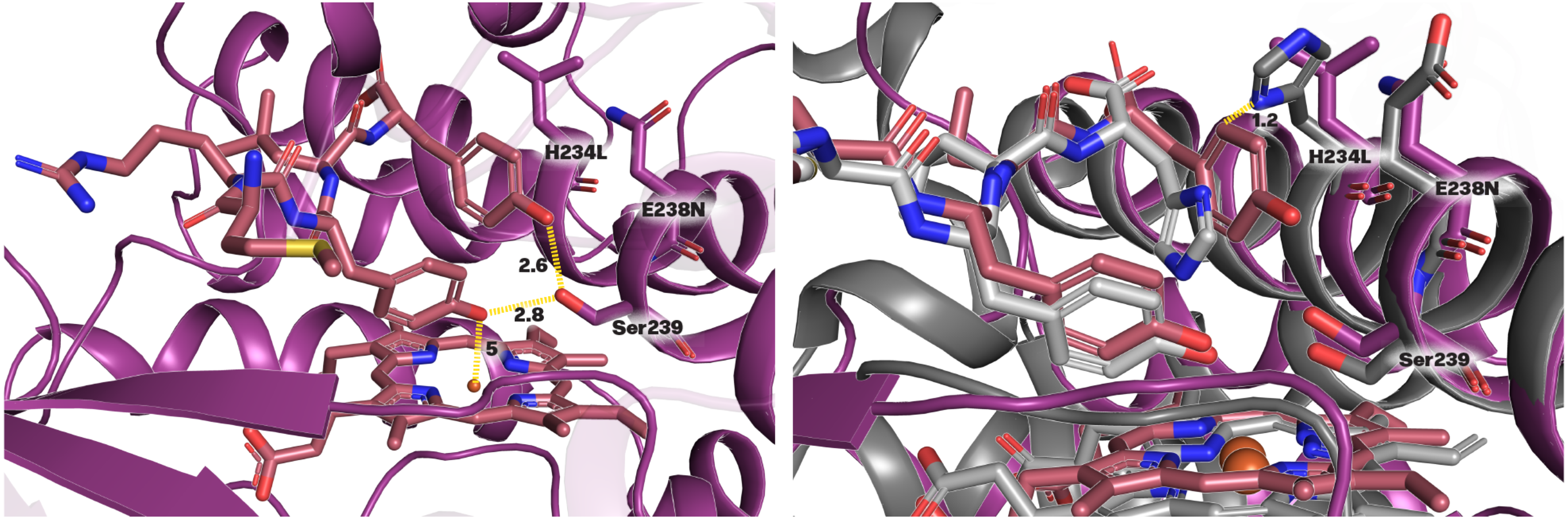

Supporting Figure S29. Investigation of the binding of 3 to Blt-M3 using MD simulations. Left – hydrogen bond network within the $P450_{Blt}$ variant *Blt-M3* (shown in purple) highlighting the hydroxyl group of Ser239 in the I-helix, which bridges the two tyrosine phenol moieties of the MRYLY substrate 3 (biarylitide and heme, both in rose). Right – superposition of the structure of wildtype $P450_{Blt}$ (PDB code 8U2M,[11] depicted in dark grey with biarylitide MRYLH and heme in light grey) with the *Blt-M3* model (shown in purple with the biarylitide MRYLY and heme in rose). This demonstrates how the H234L mutation allows the C-terminal aryl moiety to move closer to the I-helix, forming van der Waals contacts with the H234L sidechain. This contrasts with His234, which would sterically obstruct the binding of the C-terminal aryl ring in this orientation.

Supporting References

1. Zhao, Y., Marschall, E., Treisman, M., McKay, A., Padva, L., Crüsemann, M., Nelson, D. R., Steer, D. L., Schittenhelm, R. B., Tailhades, J., and Cryle, M. J. (2022) Cytochrome P450Blt Enables Versatile Peptide Cyclisation to Generate Histidine- and Tyrosine-Containing Crosslinked Tripeptide Building Blocks, *Angew. Chem. Int. Ed. 61*, e202204957.
2. Pogostin, B. H., Malmendal, A., Londergan, C. H., and Åkerfeldt, K. S. (2019) pKa Determination of a Histidine Residue in a Short Peptide Using Raman Spectroscopy, *Molecules 24*, 405.
3. Hansen, M. H., Keto, A., Treisman, M., Sasi, V. M., Coe, L., Zhao, Y., Padva, L., Hess, C., Leichthammer, V., Machell, D. L., Schittenhelm, R. B., Jackson, C. J., Tailhades, J., Crüsemann, M., De Voss, J. J., Krenske, E. H., and Cryle, M. (2024) Structural insights into a sidechain crosslinking biarylitide P450 from RiPP biosynthesis, *ACS Catal. 14*, 812–826.
4. Greule, A., Izoré, T., Iftime, D., Tailhades, J., Schoppet, M., Zhao, Y., Peschke, M., Ahmed, I., Kulik, A., Adamek, M., Goode, R. J. A., Schittenhelm, R. B., Kaczmarski, J. A., Jackson, C. J., Ziemert, N., Krenske, E. H., De Voss, J. J., Stegmann, E., and Cryle, M. J. (2019) Kistamicin biosynthesis reveals the biosynthetic requirements for production of highly crosslinked glycopeptide antibiotics, *Nat. Commun. 10*, 2613.
5. Brieke, C., and Cryle, M. J. (2014) A Facile Fmoc Solid Phase Synthesis Strategy To Access Epimerization-Prone Biosynthetic Intermediates of Glycopeptide Antibiotics, *Org. Lett. 16*, 2454-2457.
6. Tailhades, J., Schoppet, M., Greule, A., Peschke, M., Brieke, C., and Cryle, M. J. (2018) A route to diastereomerically pure phenylglycine thioester peptides: crucial intermediates for investigating glycopeptide antibiotic biosynthesis, *Chem. Commun. 54*, 2146-2149.
7. (2021) Schrödinger Release 2021-3: Gilde, Schrödinger, New York, NY.
8. (2021) Schrödinger Release 2021-3: Maestro, Schrödinger, New York, NY.
9. (2021) Schrödinger Release 2021-3: LigPrep, Schrödinger, New York, NY.
10. (2021) Schrödinger Release 2021-3: BioLuminate, Schrödinger, New York, NY.
